# TNF Production or TNFR2 Expression Characterize Distinct States of Regulatory T Cells that Cooperate in Treg Expansion in Cancer and Chronic Inflammation

**DOI:** 10.1002/eji.70062

**Published:** 2025-09-21

**Authors:** Gloria Tucci, Ilenia Pacella, Alessandra Pinzon Grimaldos, Alessandra Rossi, Ilenia Cammarata, Marta Zagaglioni, Giovanna Peruzzi, Valentina Tirelli, Massimo Sanchez, Giuseppe Pietropaolo, Francesca Sozio, Annalisa Del Prete, Valerio Licursi, Vincenzo Barnaba, Silvia Piconese

**Affiliations:** ^1^ Department of Translational and Precision Medicine Sapienza University of Rome Rome Italy; ^2^ Department of Surgery, Sapienza University of Rome Rome Italy; ^3^ Neuroimmunology Unit IRCCS Fondazione Santa Lucia Rome Italy; ^4^ Centre For Life Nano‐ & Neuro‐Science Fondazione Istituto Italiano Di Tecnologia (IIT) Rome Italy; ^5^ Core Facilities, Istituto Superiore Di Sanità Rome Italy; ^6^ IRCCS Neuromed Pozzilli Italy; ^7^ Department of Molecular Medicine Sapienza University of Rome Rome Italy; ^8^ Department of Molecular and Translational Medicine University of Brescia, Italy; IRCCS Humanitas Research Hospital Milan Italy; ^9^ Institute of Molecular Biology and Pathology (IBPM), National Research Council (CNR) of Italy c/o Department of Biology and Biotechnology “C. Darwin” Sapienza University of Rome Italy; ^10^ Department of Clinical Internal, Anesthesiological and Cardiovascular Sciences Sapienza University of Rome Rome Italy; ^11^ Laboratory Affiliated to Istituto Pasteur Italia – Fondazione Cenci Bolognetti Rome Italy

**Keywords:** oxidative stress, TNFR2, TNF, Treg, tumor microenvironment

## Abstract

TNF is a pleiotropic cytokine with immunomodulatory functions mediated by its interaction with the receptor TNFR2, highly expressed by Tregs. However, Tregs can also produce TNF, and an autocrine TNF‐TNFR2 loop has been proposed. Here, we describe that both human and mouse Tregs produce TNF in physiological conditions, in several mouse organs, and in mouse models of chronic inflammation and cancer. However, TNF production and TNFR2 expression are differentially distributed: indeed, TNFR2^+^ and TNFR2^−^ Treg subsets are, respectively, poor and strong TNF producers. The two subsets of TNFR2^+^ and TNFR2^−^ Tregs partially maintain their different ability to produce TNF when separately stimulated ex vivo. However, when cocultured, the TNFR2^+^ cells greatly outnumber the TNFR2^−^ counterpart and induce in TNFR2^−^ cells the upregulation of Foxp3 and TNFR2, in association with the transfer of cytoplasmic material. Functionally, TNFR2^+^ Tregs display superior suppressive activity and survival in vitro, both related to an improved resistance to oxidative stress. Overall, our data indicate that Tregs exist in two states, respectively committed to TNF production or TNF sensing through TNFR2, which cooperate in promoting the suppressive function of the whole Treg pool.

## Graphical abstract

Tregs exist in two functional states: TNF‐producing Tregs, which are expanded in mouse models of chronic inflammation and cancer, and TNFR2‐expressing Tregs, which dominate and transmit their phenotype to TNF+ Tregs. The two states are characterized by distinct phenotypic and functional features. Created in BioRender. Piconese, S. (2025) https://BioRender.com/5cldomx.

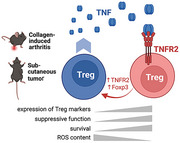

## 1 Introduction

Regulatory T cells (Tregs) are a subset of CD4 T lymphocytes characterized by the expression of Foxp3. They play a crucial role in maintaining immune and tissue homeostasis. Dysregulation in Treg numbers or functions has been observed across a wide range of pathological conditions, including autoimmune diseases and cancer. Multiple cytokine receptors influence the balance between Treg expansion and contraction [1], with tumor necrosis factor (TNF) drawing particular attention for its significant role in this process.

TNF is a pleiotropic cytokine with strong proinflammatory functions. However, multiple lines of evidence suggest that it also plays immunosuppressive roles, which may seem counterintuitive [2]. A key regulatory function of TNF involves stimulating the proliferation, effector activity, and stability of both mouse and human Tregs [3, 4, 5]. Notably, Tregs predominantly express high levels of the tumor necrosis factor receptor 2 (TNFR2) [6]. TNF stimulation induces the coordinated expression of other members of the TNFR superfamily, and their signaling activates a molecular effector program in Tregs that is mediated by NF‐κB activation [7, 8]. Previously, we demonstrated that, in vitro, TNF strongly promotes the proliferation of human Tregs, which exhibit an activated phenotype and possess enhanced suppressive abilities [9]. Additionally, we provided evidence of crosstalk between autoreactive, unconventional naive‐like CD8^+^ T cells producing TNF, and TNFR2^+^ Tregs. This interaction enables the expansion of Tregs, which in turn suppress the differentiation of the CD8^+^ T cells in rheumatoid arthritis (RA) patients responding to anti‐TNF therapy [10]. This data supports prior findings that anti‐TNF treatments can lead to the expansion of TNFR^+^ Tregs in RA patients who benefit from this therapy [11].

TNF plays multiple roles in cancer, including some that are opposing, due to the complex interactions among the cells that produce and respond to this cytokine within the tumor microenvironment [12]. It remains unclear whether the protumor effects of TNF are primarily due to its immunosuppressive properties, such as promoting Treg expansion, and this question warrants further investigation [2]. Supporting this possibility, some studies have shown that, in cancer patients, the proportion of circulating Tregs positively correlates with serum TNF levels [13]. Additionally, in experimental models, blocking TNF or TNFR2 in vivo reduces tumor growth while limiting effector Tregs [13]. Previous studies demonstrated that TNFR2 co‐stimulation promotes glycolysis and lipid biosynthesis in human Tregs [14, 15]. Therefore, the TNF/TNFR axis likely plays a crucial role in sustaining Treg‐mediated immune suppression within the tumor microenvironment.

Many cell types, especially proinflammatory cells like macrophages and activated T cells, can produce TNF. This cytokine is initially synthesized as a transmembrane trimer (tmTNF), which can be cleaved by the TNF converting enzyme (TACE) to release a soluble form (sTNF). The signaling pathways of TNF are complex and involve several factors: (i) the two forms of TNF have different capacities to bind to TNFR1 and TNFR2, with tmTNF primarily binding to TNFR2, potentially leading to distinct functions; (ii) soluble forms of TNFR1 and TNFR2 can be released through proteolytic cleavage or alternative splicing, allowing them to compete for ligand binding; (iii) the interaction between tmTNF and TNFR2 can trigger both forward and reverse signaling; (iv) depending on the stimulus and context, TNF may exert autocrine or paracrine effects; (v) autocrine signaling of TNF can also occur [16, 17].

Recent studies have shown that human Tregs can produce TNF, suggesting a possible role for autocrine TNF stimulation in their expansion. Circulating Tregs constitutively express tmTNF and release sTNF when strongly stimulated in vitro [18, 19]. Notably, TNF is the only cytokine produced by human umbilical cord‐derived Tregs expanded in vitro [20]. Neutralizing TNF or blocking TNFR2 reduces Treg proliferation even in the absence of external TNF sources [18, 21].

Tregs capable of producing TNF have also been identified in pathological conditions such as acute viral hepatitis, where their presence correlates positively with the severity of liver damage. These Tregs display an epigenetic signature indicative of lineage stability [22]. Moreover, TNF‐producing Tregs have been associated with a Th17‐like phenotype in various pathological contexts, including cancer [18, 22, 23]. A significant reduction in Treg percentages has been observed in both the thymus and spleen of TNF‐knockout (KO) mice compared with wild‐type (WT) mice [24], although the key sources of TNF in this setting were not examined. It remains unclear whether autocrine or paracrine TNF loops are involved in Treg expansion during normal physiology or in conditions like tumor progression. Addressing this question could improve our understanding of TNF's complex and seemingly paradoxical roles in cancer progression and may serve as a foundation for developing more targeted immunotherapies [2, 12].

## 2 Results

### 2.1 Tregs, Especially the TNFR2‐Negative, Produce TNF in Physiological Conditions

While several studies have demonstrated TNF production by human Tregs, particularly under pathological conditions [18, 19, 22, 23], there is limited information regarding TNF expression by murine Tregs. To address this gap, we conducted a systematic analysis of total TNF expression using intracellular flow cytometry. We examined lymphocytes isolated from various lymphoid (inguinal and mesenteric lymph nodes, spleen) and nonlymphoid organs (lung, liver, bone marrow) of naive adult mice, following ex vivo stimulation with PMA and ionomycin.

**FIGURE 1:**
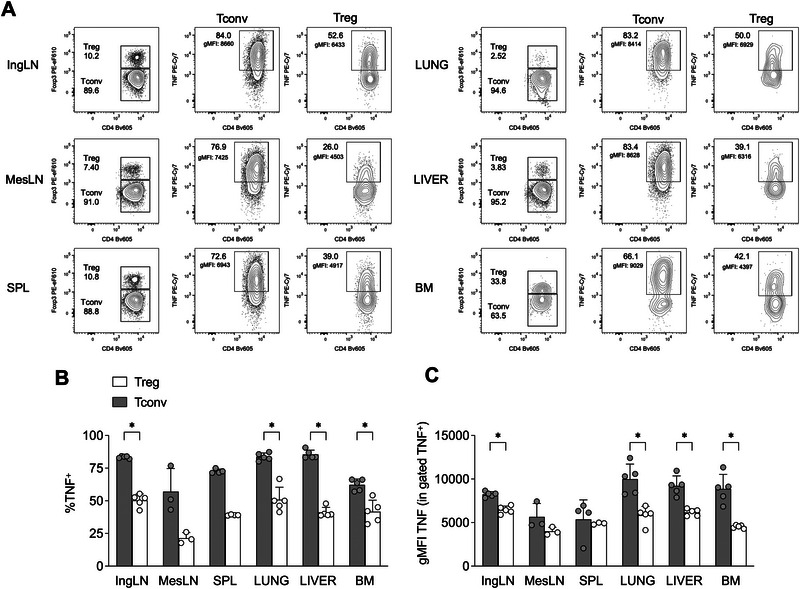
Tregs produce TNF in several organs in naïve mice. (A) Representative flow cytograms showing percentages of TNF^+^ cells, and geometric mean fluorescence intensity (gMFI) of TNF in gated TNF^+^ cells, in Treg and Tconv from the inguinal lymph node (IngLN), mesenteric lymph node (MesLN), spleen (SPL), lung, liver, and bone marrow (BM), of naïve C57BL/6 mice. (B,C) Cumulative analysis showing percentages of TNF^+^(B), and gMFI of TNF in gated TNF^+^ cells (C), in Treg and Tconv of the indicated organs. Data are from one representative of two independent experiments, each performed with 3–5 mice. Bars represent means and SD. *p < 0.05, by multiple Mann–Whitney test with Holm–Sidak correction for multiple comparisons.

Our results showed that Tregs produce TNF across all examined tissues, although at lower frequencies and with reduced intensities compared with conventional T cells (Tconvs) (Figure 1; Figure S1). Consistent with previous findings [18], we observed that human circulating Tregs from healthy adult donors also produce TNF upon ex vivo restimulation, again at levels lower than Tconvs. Notably, the FOXP3^lo^ CD45RA^‐^ non‐Treg subset [25] tended to exhibit the highest TNF expression, while resting (FOXP3^lo^ CD45RA^+^) and activated (FOXP3^hi^ CD45RA^−^) Tregs displayed comparable TNF levels (Figure S2).

Since Tregs are known to highly express TNFR2 [6], we examined TNFR2 expression in Tregs (and Tconvs as controls) either freshly isolated from the spleens of naive mice or following in vitro polyclonal stimulation. Ex vivo, approximately one‐third of Tregs expressed TNFR2, while Tconvs showed no detectable expression (Figure S3A).

Upon activation, both Tregs and Tconvs upregulated TNFR2 and produced TNF; however, Tregs exhibited significantly higher levels of TNFR2 expression (Figure S3B). When a TNF‐neutralizing antibody was added to the cultures, TNFR2 induction and proliferation remained unaffected in Tconvs but were markedly inhibited in Tregs (Figure S3C,D). These findings suggest that Treg‐derived TNF contributes to their own expansion and amplifies this effect by increasing TNFR2 expression.

**FIGURE 2:**
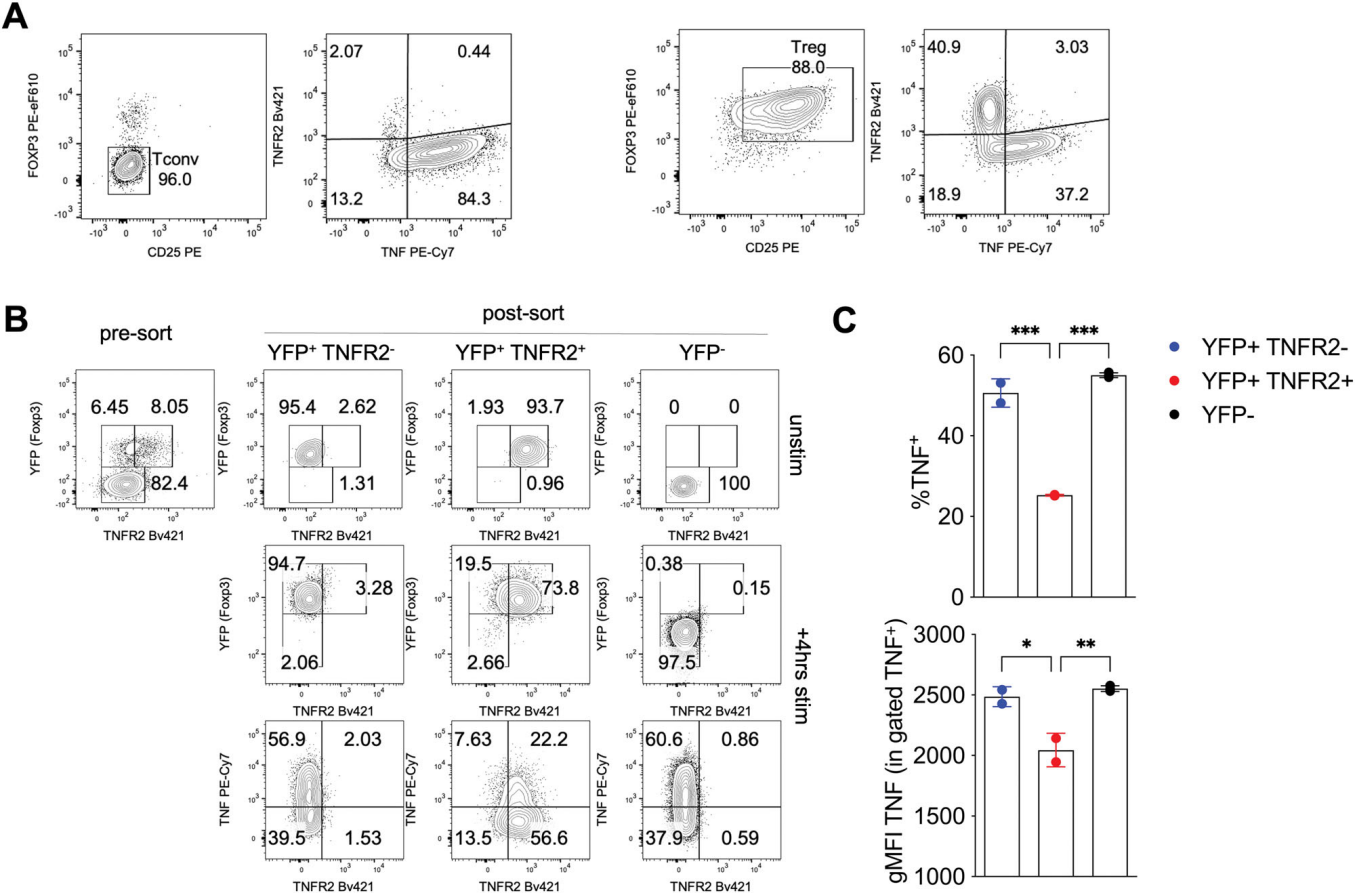
TNF and TNFR2 are differentially distributed in Tregs. (A) Tregs and Tconvs were immunomagnetically enriched as CD4^+^CD25^+^ and CD25^−^, respectively, from the spleens of C57BL/6 mice and restimulated in vitro 4 h before analysis of surface TNFR2 and intracellular TNF. (B, C) TNFR2^+^ and TNFR2^−^ Tregs and Tconvs were sorted as YFP^+^ and YFP^−^ CD4 T cells, respectively, from the spleens of *Foxp3* ^CreYFP^ mice and restimulated in vitro 4 h before analysis of surface TNFR2 and intracellular TNF. (B) Representative cytograms showing the purity of sorted cells, and TNF/TNFR2 expression in unstimulated and stimulated cells. (C) Cumulative analysis showing percentages of TNF^+^, and gMFI of TNF in gated TNF^+^ cells, in the indicated subpopulation (each tested in 2–3 replicates). Data are from one representative of two independent experiments, each performed with CD4 T cells from the pooled splenocytes of three mice. Bars represent means and SD. *p < 0.05, **p < 0.01, ***p < 0.001, by one‐way ANOVA.

Next, we investigated whether this TNF‐TNFR2 feedback loop is autocrine or paracrine. Specifically, we examined whether TNF and TNFR2 were coexpressed within the same Treg subpopulations. While Tconvs displayed high TNF production but negligible TNFR2 expression, Tregs contained two distinct subpopulations: one that expressed TNFR2 and another that produced TNF (Figure 2A).

We observed that the 4 h stimulation with PMA‐ionomycin, required for analyzing TNF, significantly decreased the detection of TNFR2 on the cell surface (Figure S4). A possible explanation for this observation is TNFR2 shedding from the cell surface, which is known to occur upon activation in mouse and human Tregs [26]. To explore TNF and TNFR2 expression in Tregs, we sorted TNFR2‐positive and TNFR2‐negative Tregs from *Foxp3* ^CreYFP^ reporter mice and then restimulated these cells to assess their TNF content. Even in this setting, we confirmed that TNFR2^+^ Tregs contained lower levels of TNF, both in terms of the frequency of TNF‐positive cells and the fluorescence intensity within positive cells (Figure 2B,C).

Kinetic analysis showed that TNF production began as early as 30 min after restimulation and gradually increased across all cell types, with TNFR2^+^ Tregs reaching lower overall levels of TNF (Figure S5A). Additionally, soluble TNF was detected in cell culture supernatants by ELISA, with the most pronounced differences between TNFR2^+^ and TNFR2^−^ Tregs occurring between 1 and 2 h poststimulation (Figure S5B).

Overall, these findings demonstrate that, in naive mice, Tregs not only express TNFR2 but also produce TNF. However, the expression of these two molecules appears to be differentially distributed within individual cells.

### 2.2 TNF‐Producing Tregs Expand in Cancer and in Chronic Arthritis

**FIGURE 3:**
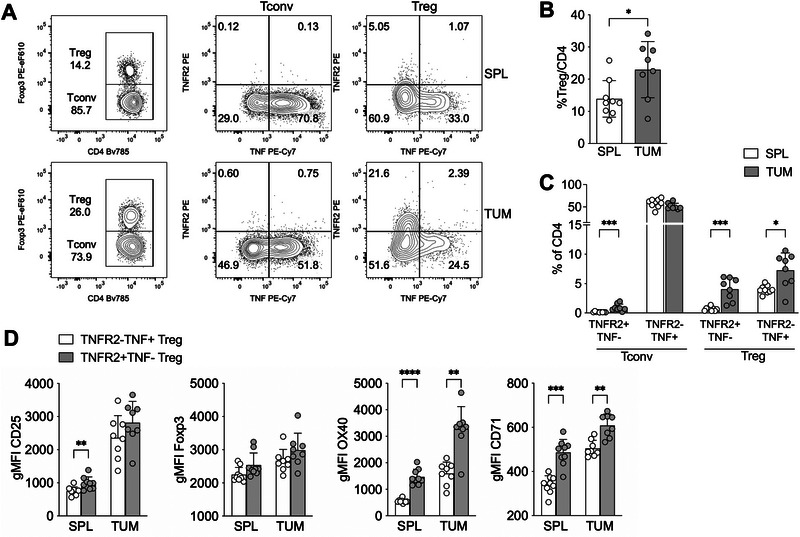
Both TNF^+^ and TNFR2^+^ Tregs are expanded in tumors. (A) Representative cytograms showing Treg and Tconv frequencies and TNF/TNFR2 expression in spleen (SPL) and tumor (TUM) samples, from mice bearing MC38 subcutaneous tumors. (B–D) Cumulative analysis showing percentages of Tregs among CD4 (B), TNFR2^+^TNF^−^ and TNFR2^−^TNF^+^ Treg and Tconv among CD4 T cells (C), and the gMFI of the indicated markers (D), in SPL and TUM samples of MC38 tumor‐bearing mice (*n *= 9). Bars represent means and SD. *p < 0.05, **p < 0.01, ***p < 0.001, ****p < 0.0001, by multiple Mann–Whitney test with Holm–Sidak correction for multiple comparisons.

To assess whether pathological conditions influence TNFR2^+^ and TNF^+^ Tregs, we analyzed their frequency and phenotype within a tumor microenvironment, which is a context characterized by Treg expansion. In both MC38 (Figure 3A,B) and 18.5 (Figure S6A,B) tumor models, the frequency of Tregs was higher at the tumor site compared with the spleen, as expected. Notably, both the TNFR2^+^TNF^‐^ and TNFR2^−^TNF^+^ Treg subsets contributed to the increased Treg population within tumors (Figure 3C; Figure S6C).

In both MC38 (Figure S7) and 18.5 (not shown) models, tumor‐infiltrating Tregs and Tconvs displayed bright intracellular expression of total TNF and TNFR2, respectively. However, TNFR1 and transmembrane (tm) TNF were weakly detected in both Tconvs and Tregs. Phenotypically, TNFR2^+^TNF^−^ Tregs expressed higher levels of CD25 in the spleen, and of OX40 and CD71 in both spleen and tumor, compared with TNFR2^−^TNF^+^ Tregs. Only a trend toward higher Foxp3 expression was observed in TNFR2^+^TNF^−^ Tregs (Figure 3D).

**FIGURE 4:**
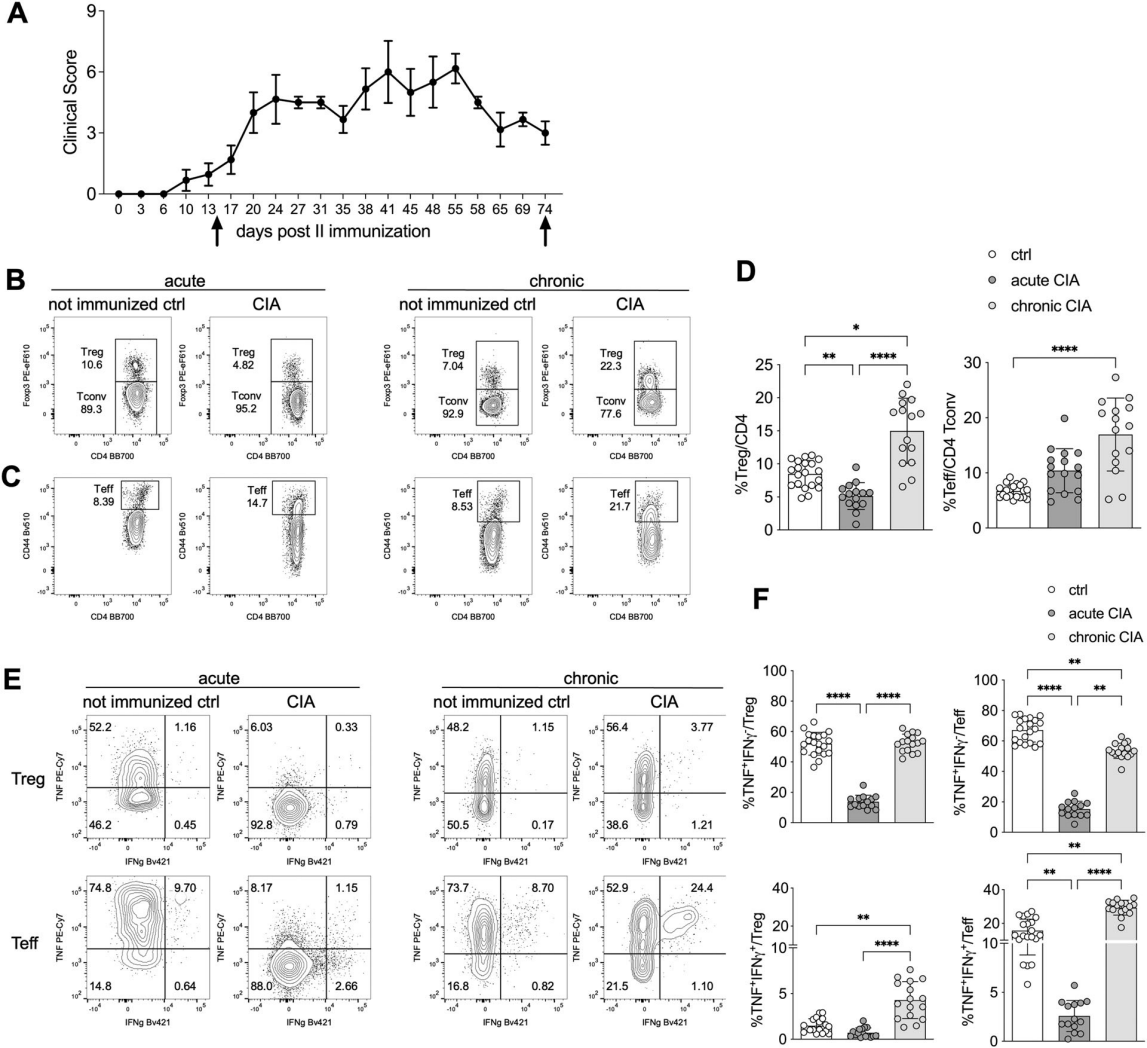
Treg recovery is associated with TNF production in chronic CIA. (A) C57BL/6 females (*n *= 16) were immunized with chicken collagen type II as described in the Methods section. Mice were sacrificed after 2 weeks (acute CIA, *n* = 8) or 10 weeks (chronic CIA, *n* = 8), after the second immunization, and two draining lymph nodes per mouse were collected. Arrows indicate the two time points of analysis. As control, inguinal lymph nodes were harvested from nonimmunized sex‐ and age‐matched mice at the two time points (ctrl, *n* = 10). (B, C) Representative plots showing Treg and Tconv frequencies in gated CD4 T cells (B), and the percentage of CD44 Teff in gated Tconv (C). (D) Analysis of Treg and Teff percentages in lymph nodes of mice with acute or chronic CIA, compared with naïve controls (ctrl). (E) Representative plots of intracellular TNF and IFNγ in Treg and Teff (identified as CD44^+^ Tconv) in the indicated samples. (F) Analysis of the percentages of TNF^+^ IFNγ^+^/^− ^cells in gated Treg or Teff. In all plots, bars represent means and SD. Each dot is a single lymph node. Statistically significant outliers were identified with the Rout method and removed from the analysis. **p < 0.01, ****p < 0.0001, by Kruskal–Wallis test with Dunn's multiple comparison.

TNF is widely recognized for its role in the pathogenesis of arthritis in both patients and mouse models, and TNF‐blocking therapies remain among the first‐line treatments for rheumatoid arthritis [27]. However, TNF also has several immunoregulatory functions [28]. To investigate whether Tregs are a source of TNF during the progression of arthritis, we analyzed their activation status and TNF production in the draining lymph nodes of mice with either acute (2 weeks post‐second immunization) or chronic (10 weeks post‐immunization) collagen‐induced arthritis (CIA) (Figure 4A).

During the acute phase, Treg frequencies decreased, but they increased again during the chronic phase, coinciding with a progressive accumulation of CD44^hi^ effector T cells (Teffs) (Figure 4B–D). When measuring cytokine production after ex vivo restimulation—specifically TNF in combination with IFNγ—both Teffs and Tregs showed a high proportion of cells producing only TNF, which significantly declined in the acute phase and recovered in the chronic phase. Conversely, cells producing both TNF and IFNγ were predominantly found among Teffs and only modestly increased among Tregs during the chronic phase (Figure 4E,F). These data suggest that during the chronic phase of arthritis, when inflammation is thought to be resolving, Tregs expand and produce high levels of TNF.

### 2.3 TNF Expression is Both Transcriptionally and Posttranscriptionally Regulated in TNFR2^+^ Tregs

The observation that TNFR2^+^ Tregs produce relatively little TNF prompted us to explore the mechanisms underlying this regulation. Since TNF expression can be controlled at both transcriptional and posttranscriptional levels [29, 30], we examined the combined mRNA and protein levels of TNF in sorted TNFR2^+^ and TNFR2^−^ Tregs using a flow cytometry‐based hybridization assay.

In TNFR2^−^ Tregs, similar to Tconvs, most cells positive for TNF mRNA also expressed TNF protein. In contrast, a higher proportion of TNFR2^+^ Tregs expressed TNF mRNA but not TNF protein, suggesting a partial blockade in TNF translation (Figure S8A). Further, real‐time qPCR analysis performed 1.5 h post stimulation showed that TNF mRNA levels in sorted TNFR2^+^ Tregs were significantly reduced (Figure S8B). Meanwhile, TNF protein released into the supernatant was completely absent (Figure S8C), indicating that both transcriptional and posttranscriptional mechanisms contribute to TNF regulation in TNFR2^+^ Tregs.

Since miR146a is known to play key roles in Treg function [31] and to modulate TNF expression in other cell types [32], we investigated whether this microRNA might regulate TNF production in TNFR2^+^ Tregs. A flow cytometry‐based hybridization assay revealed that miR146a was more abundant in TNFR2^+^ Tregs (Figure S9A,B). Interestingly, we identified among TNFR2^+^ Tregs a population of miR146a^high^ TNF^‐^ cells (Figure S9C).

Using real‐time qPCR, we confirmed that miR146a was substantially more expressed in TNFR2^+^ Tregs compared with TNFR2^−^ Tregs, while miR146b was not detected in any subpopulation (Figure S9D). When miR146a was silenced with an antagomir targeting its 5' arm (Figure S9E), there was no significant change in the percentage of TNF^+^ cells; however, the intensity of TNF staining within TNF^+^ cells was slightly but significantly increased in silenced TNFR2^+^ Tregs (Figure S9F,G). Conversely, an antagomir targeting the 3' arm of miR146a failed to silence the microRNA (and actually increased its expression) and did not affect TNF production (Figure S10).

Altogether, these data suggest that miR146a‐5P may modestly contribute to the posttranscriptional regulation of TNF in TNFR2^+^ Tregs.

### 2.4 TNFR2^+^ Tregs Dominate Over TNFR2^−^ Tregs and Display Stronger Survival and Suppression

While TNFR2^+^ and TNFR2^−^ Tregs are not necessarily distinct lineages, they likely represent two functional states that can still exhibit differences in (i) functional stability, (ii) interaction via TNF‐TNFR2, (iii) phenotypic profile, (iv) suppressive capacities, and (v) gene expression signature.

**FIGURE 5:**
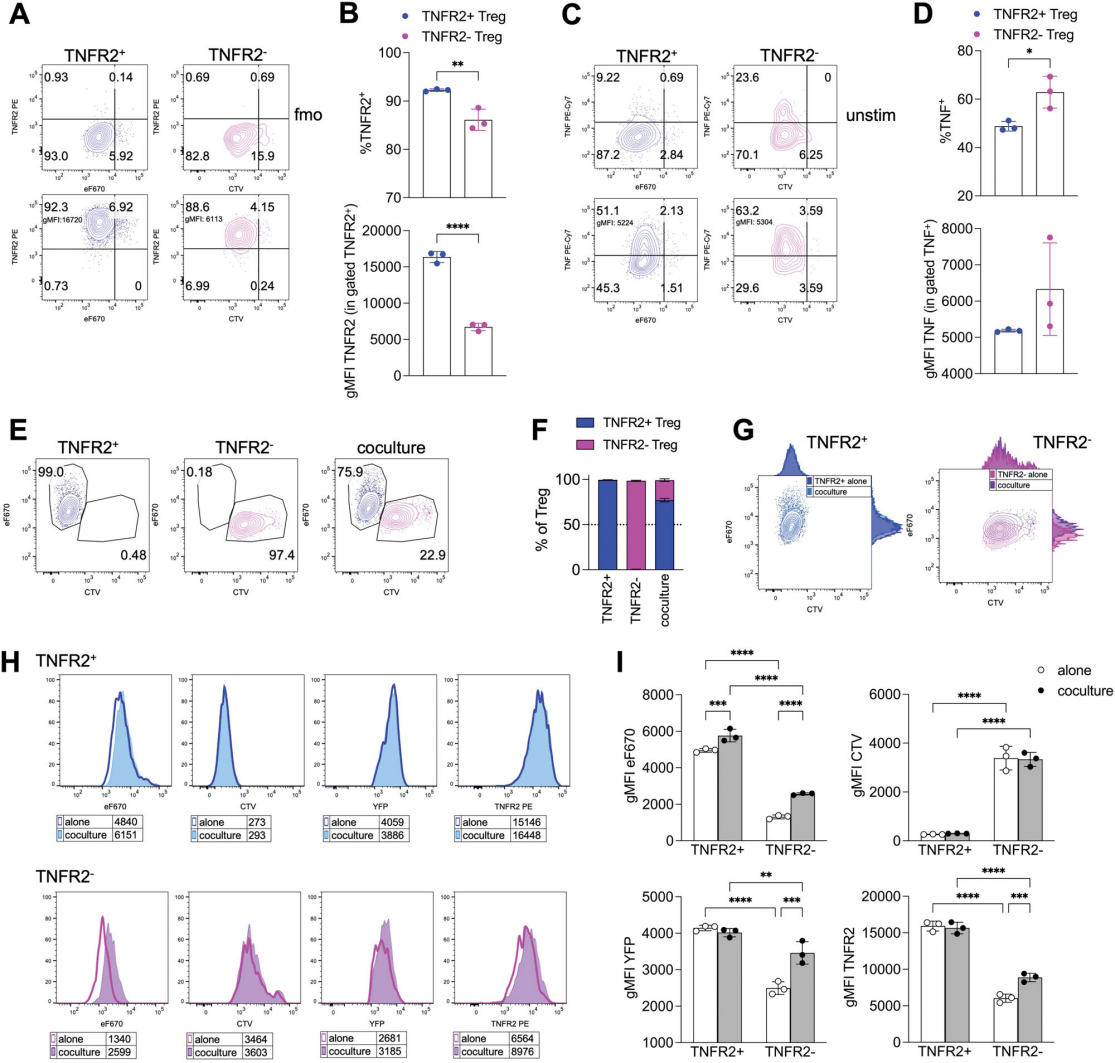
TNFR2^+^ and TNFR2^−^ Treg maintain their diversity, while TNFR2^−^ are more activated when cocultured with TNFR2^+^. (A–D) TNFR2^+^ and TNFR2^−^ Tregs were sorted from the spleens of *Foxp3* ^CreYFP^ mice, respectively labeled with eF670 (blue) or CTV (purple) proliferation dyes, cultured 3–4 days with coated aCD3/aCD28 in the presence of IL‐2, and then analyzed by flow cytometry (after restimulation in vitro for 4 h for the analysis of intracellular TNF). (A) TNFR2 expression against each proliferation dye in each cell type. (B) Percentages of TNFR2^+^ cells, and geometric mean fluorescence intensity (gMFI) of TNFR2 in gated TNFR2^+^ cells, in the indicated cell types. (C) TNF expression against each proliferation dye in each cell type. (D) Percentages of TNF+ cells, and geometric mean fluorescence intensity (gMFI) of TNF in gated TNF^+^ cells, in the indicated cell types. *p < 0.05, **p < 0.01, ****p < 0.0001, by Student's *t*‐test. (E–I) TNFR2^+^ and TNFR2^−^ Tregs were seeded alone or cocultured at 1:1 ratio. (E, F) Representative cytograms and percentages of each cell type in the indicated conditions. (G) Contour plot overlays with adjunct histograms showing the fluorescence of the two proliferation dyes in TNFR2^+^ and TNFR2^−^ Tregs, either alone or cocultured. (H, I) Representative histogram overlays and analysis of the gMFI of the indicated dyes and markers in TNFR2^+^ and TNFR2^−^ Tregs, either alone or cocultured. **p < 0.01, ***p < 0.001, ****p < 0.0001, by two‐way ANOVA. Data are from one experiment representative of three. Each condition was tested in triplicate.

First, we assessed whether sorted TNFR2^+^ and TNFR2^−^ Tregs maintained their key features after polyclonal stimulation in vitro for 4 days. Both subsets proliferated effectively, upregulated TNFR2, and produced TNF. Nonetheless, they preserved significant differences in expression levels of TNFR2 (Figure 5A,B) and TNF (Figure 5C,D).

Second, to explore their interactions, we fluorescently labeled and co‐cultured TNFR2^+^ and TNFR2^−^ Tregs at a 1:1 ratio. Remarkably, by the end of culture, TNFR2^+^ Tregs greatly outnumbered the TNFR2^−^ subset (Figure 5E,F). To determine if this advantage was due to higher proliferation, we analyzed the dilution of the respective dyes eF670 and CTV. We observed that, when co‐cultured, TNFR2^−^ Tregs acquired some fluorescence from the eF670 dye derived from TNFR2^+^ cells, while the reverse did not occur (Figure 5G). Moreover, TNFR2^+^ Tregs proliferated slightly less when co‐cultured compared with alone, suggesting their dominance was not solely due to proliferative advantage. Conversely, TNFR2^−^ Tregs showed no difference in proliferation when cultured alone or with TNFR2^+^ cells but gained eF670 fluorescence and increased expression of Foxp3‐YFP and TNFR2 upon co‐culture (Figure 5H,I). These findings indicate that, when cultured with TNFR2^+^, TNFR2^−^ Tregs acquired a TNFR2^+^ and Foxp3^high^ phenotype.

**FIGURE 6:**
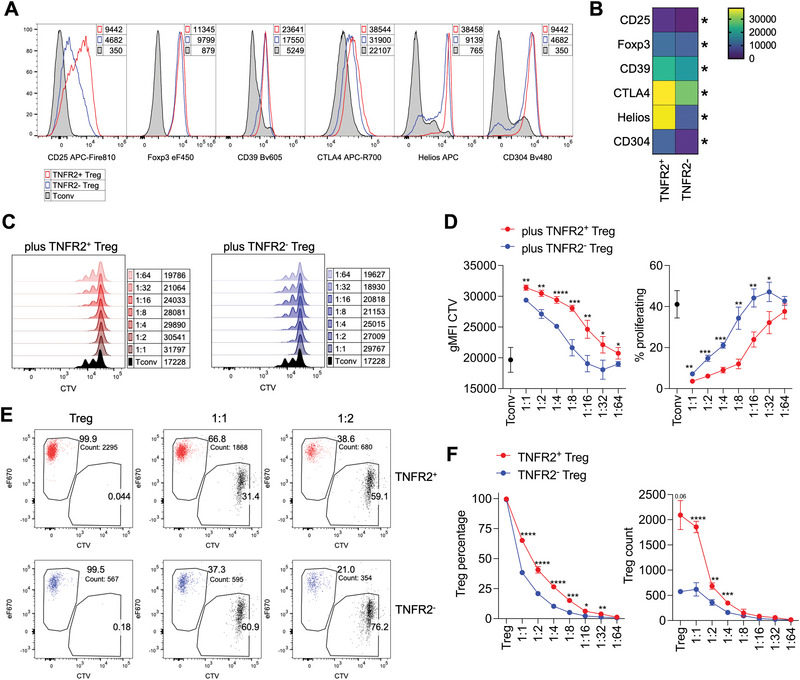
*TNFR2^+^* Tregs display superior suppressive function and survival in vitro. (A, B) TNFR2^+^ and TNFR2^−^ Tregs were sorted from the spleens of *Foxp3* ^eGFP‐Cre‐ERT2^ mice, and their phenotype was freshly analyzed through flow cytometry. The plots display histogram overlays (A) and a heatmap of the z‐score (B), calculated from means and SD of gMFI of each marker in Tconvs, TNFR2^+^, and TNFR2^−^ Tregs (*n* = 4). Data are from one experiment representative of two. *p < 0.05, by Mann–Whitney test. (C–F) Tconv were sorted as YFP^−^ cells, labeled with the CTV proliferation dye, and cultured in vitro either alone or at scaled ratios with eF670‐labeled TNFR2^+^ or TNFR2^−^ Tregs. (C) Histogram overlays showing CTV dilution and respective gMFI in gated Tconv cultured alone (black) or cocultured with TNFR2^+^ (red) or TNFR2^−^ (blue) Tregs in the indicated conditions. (D) Analysis of the gMFI of CTV and of the percentage of proliferating cells in gated Tconv in the indicated conditions. (E, F) Representative cytograms and cumulative analysis showing the percentages and the counts of Treg in each coculture condition. Data are from one representative of two independent experiments. Each condition was tested in 2–6 replicates. *p < 0.05, **p < 0.01, ***p < 0.001, ****p < 0.0001, by unpaired *t*‐test with Holm–Sidak correction for multiple comparisons.

Third, we analyzed the expression of markers associated with Treg stability and function, such as CD25, Foxp3, CD39, CTLA4, Helios, and Nrp1 (CD304). We found that these molecules were all significantly more expressed in TNFR2^+^ Tregs than in TNFR2^−^ Tregs (Figure 6A,B). Additionally, TNFR2^+^ Tregs produced lower amounts of IL‐17 and IL‐10, with no significant difference in IFNγ levels (Figure S11).

Fourth, we tested whether TNFR2^+^ and TNFR2^−^ Tregs differed in suppressive ability. Both subsets were labeled with eF670 and cultured at various ratios with CTV‐labeled responder T cells. Results showed that TNFR2^+^ Tregs were more suppressive than TNFR2^−^ Tregs (Figure 6C,D). A quantitative advantage of TNFR2^+^ Tregs may account for their increased suppressive capacity. Indeed, we observed that the percentages and absolute counts of TNFR2^+^ Tregs were higher than TNFR2^−^, at multiple ratios of co‐culture with T responders (Figure 6E,F). Such higher recovery was not explained by increased proliferation. Indeed, analysis of eF670 dye dilution indicated that TNFR2^+^ Tregs proliferated slightly less than TNFR2^−^ Tregs (Figure S12).

**FIGURE 7:**
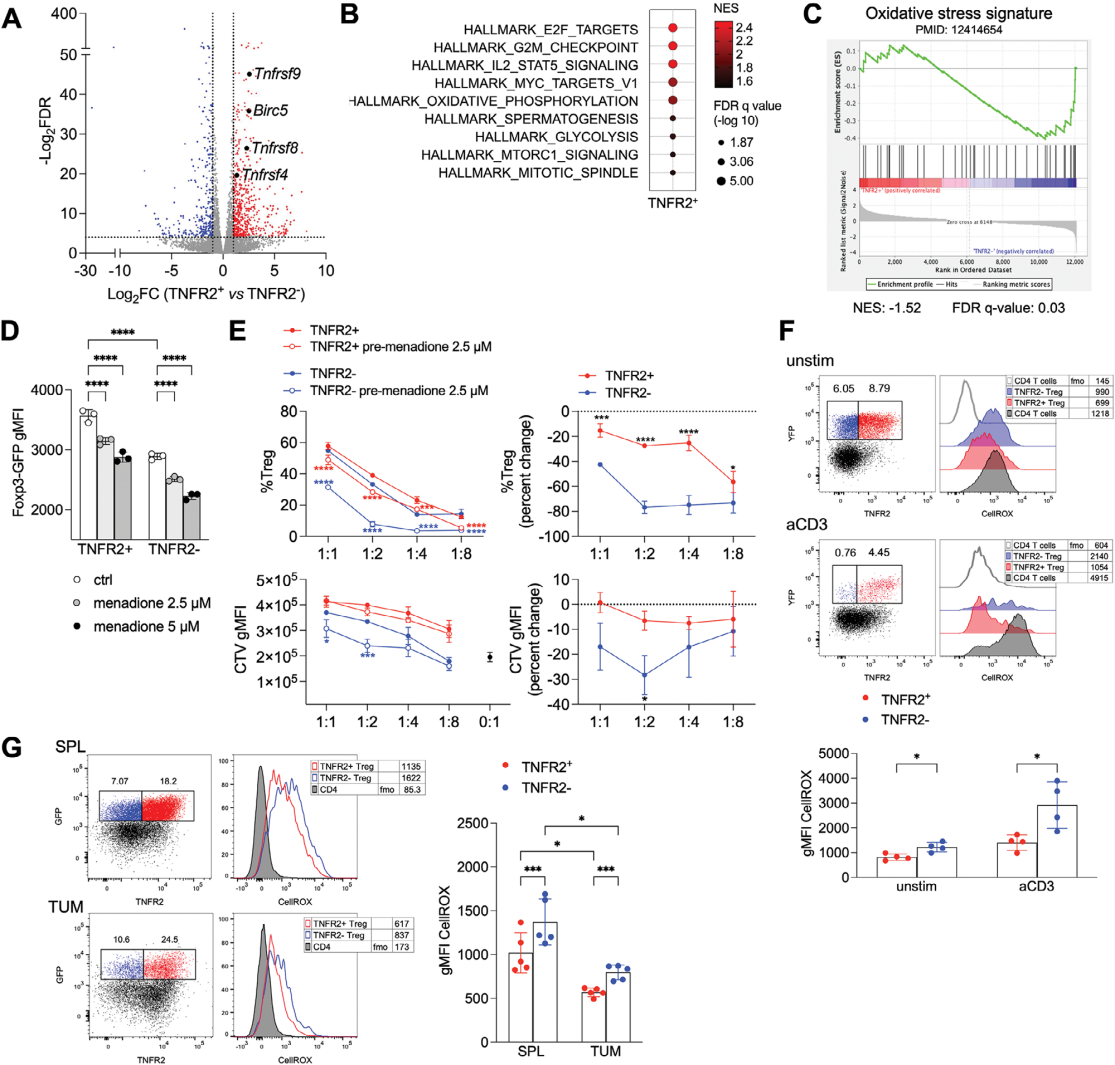
The molecular program of TNFR2^+^ Tregs supports survival and resistance to oxidative stress. (A) Volcano plot showing the fold change in gene expression by RNAseq of TNFR2^+^ versus TNFR2^−^ Treg sorted from spleens of *Foxp3* ^CreYFP^ mice (*n* = 4). Genes with FDR <0.05 and with fold change >2 (red) or <−2 (blue) are highlighted. (B) Statistically significant (FDR <0.05) results from the gene set enrichment analysis of the transcriptome of TNFR2^+^ versus TNFR2^−^ Treg in the hallmarks gene sets. (C) Gene set enrichment analysis of the transcriptome of TNFR2^+^ versus TNFR2^−^ Treg. A gene set of oxidative stress was obtained from a published signature [44]. Normalized enrichment scores (NES) and FDR q values are shown under the plot. (D) Expression of Foxp3 was estimated as the gMFI of GFP in TNFR2^+^ versus TNFR2^−^ Treg sorted from spleens of *Foxp3* ^eGFP‐Cre‐ERT2^ mice and treated for 1 h with the indicated concentrations of menadione. ****p < 0.0001, by two‐way ANOVA. (E) Suppression assay was performed with GFP^+^ TNFR2^+^ versus TNFR2^−^ Treg, either untreated or pretreated for 1 h with menadione, against CTV‐labeled Tconv. Treg percentage (as a measure of survival) and gMFI of CTV (as a measure of suppression) are shown at different Treg:Tconv ratios. Right plots represent the percentage of change between untreated and menadione‐treated cells. Each condition was tested in triplicate. Data are from one experiment representative of two. *p < 0.05, ***p < 0.001, ****p < 0.0001, by two‐way ANOVA. (F) Representative cytograms and cumulative analysis of CellROX staining in gated TNFR2^+^ or TNFR2^−^ Treg from spleens of *Foxp3* ^CreYFP^ mice, either unstimulated or stimulated with aCD3 for 18 h. Numbers indicate the geometric mean fluorescence intensity (gMFI). The fmo of CellROX as a negative control is shown. Each condition was tested in quadruplicate. Bars represent means and SD. *p < 0.05, by unpaired *t*‐test with Holm–Sidak correction for multiple comparisons. (G) Representative cytograms and cumulative analysis of CellROX staining in gated TNFR2^+^ or TNFR2^−^ Treg in unstimulated lymphocytes from spleens or tumors of *Foxp3* ^eGFP‐Cre‐ERT2^ mice (*n* = 5), previously injected with MC38 tumor cells. Numbers indicate the geometric mean fluorescence intensity (gMFI). The fmo of CellROX as a negative control is shown. Data are from one experiment representative of two. Bars represent means and SD. *p < 0.05, ***p < 0.001, by two‐way ANOVA with Geisser–Greenhouse correction and Sidak multiple comparisons test.

Fifth, bulk RNA sequencing of sorted populations revealed that TNFR2^+^ Tregs overexpressed TNF receptor superfamily members such as OX40 (*Tnfrsf4*), CD30 (*Tnfrsf8*), and 4‐1BB (*Tnfrsf9*), as well as *Birc5*, which encodes for survivin—a protein involved in T cell expansion and differentiation downstream of OX40 signaling [33, 34] (Figure 7A).

The gene signature of TNFR2^+^ Tregs was significantly enriched in profiles associated with stable and highly suppressive Treg phenotypes, including perinatal expanded Tregs [35], tissue‐resident Tregs [36], tumor‐infiltrating Tregs [37], and sepsis‐related TNFR2^+^ Tregs [38] (Figure S13). Pathways related to cell cycle, proliferation, survival, mTOR, and Myc‐dependent metabolism were prominently enriched in TNFR2^+^ Tregs (Figure 7B).

Overall, these findings indicate that TNFR2^+^ Tregs possess a stable profile and a survival advantage that confer superior suppressive capacity and a dominant influence over TNFR2^−^ Tregs.

### 2.5 Reduced ROS Content in TNFR2^+^ Tregs

Oxidative stress is a key factor influencing T cell survival [39], and Tregs particularly depend on mechanisms that protect against oxidative damage for their expansion and function [40, 41, 42, 43]. Notably, an oxidative stress‐related gene signature [44] was significantly less prominent in TNFR2^+^ Tregs (Figure 7C).

To functionally assess resistance to oxidative stress, we treated TNFR2^+^ and TNFR2^−^ Tregs with the ROS inducer menadione [42]. TNFR2^−^ Tregs expressed lower levels of Foxp3, as shown in Figure 6. Notably, treatment with menadione reduced Foxp3 expression in a dose‐dependent fashion in both cell types (Figure 7D). When Treg subsets, pretreated with menadione, were cocultured with untreated Tconvs, we observed that menadione reduced the survival of both TNFR2^−^ and TNFR2^+^ Tregs, even though this reduction was significantly more pronounced in TNFR2^−^ Tregs. This resulted in a significant decrease in the suppression potency of TNFR2^−^ Tregs (Figure 7E). These data suggest that TNFR2^+^ Tregs are more resistant to oxidative stress, preserving their survival and suppressive function.

To estimate the ability to control the intracellular ROS load, cells were stained with CellROX dye from both unstimulated and polyclonally stimulated splenocytes. We found that ROS content was significantly lower in TNFR2^+^ Tregs compared with TNFR2^−^ Tregs (Figure 7F). We also assessed ROS levels directly ex vivo from spleens and tumors of MC38 tumor‐bearing mice. Both TNFR2^+^ and TNFR2^−^ Tregs exhibited lower ROS levels within tumors compared with spleens, likely due to in vivo activation. Importantly, ROS content was significantly lower in TNFR2^+^ Tregs relative to TNFR2^−^ Tregs in both spleens and tumors (Figure 7G).

These observations underscore the importance of reduced oxidative stress in conferring functional superiority to TNFR2^+^ Tregs within immunosuppressive environments.

## 3 Discussion

Several pieces of evidence point to immunoregulatory roles of TNF, which are mostly related to its signal on Tregs through the TNFR2. Here we show that Tregs contain two subpopulations, respectively committed to TNF production and to TNFR2‐mediated TNF sensing, which both increase at the tumor site. These two subsets maintain their diversity when separately stimulated ex vivo. However, when cocultured, the TNFR2^+^ Tregs dominate over the TNFR2^−^, and even induce a stronger expression of Foxp3 and TNFR2 in the TNFR2^−^ Tregs.

TNF production by Tregs has been reported in several studies, even though with conflicting interpretations. In some pathological conditions, TNF‐producing Tregs are associated with reduced suppressive function and stability and with more severe disease [22]. Conversely, other studies suggest that Treg‐derived TNF promotes their own proliferation, suppression, and stability in vitro and in vivo [18, 19, 23]. TNF production by Tregs may have divergent outcomes, depending on whether it is produced as a single cytokine by epigenetically stable Tregs or together with other proinflammatory cytokines, such as IFNγ or IL‐17, by unstable Tregs. Highly pure, in vitro expanded, human Tregs, which are Helios^+^ and exert potent immune suppressive functions in vivo, do not produce any cytokine except TNF [20]. In a mouse model of arthritis, we observed strong TNF production by both Tconvs and Tregs during the chronic but not the acute phase of the disease. While both Tconvs and Tregs contained around 50% of single TNF producers, the double TNF‐IFNγ producers were 30% in the Tconv gate, and only around 5% in the Treg gate, in the chronic phase of CIA. This speaks in favor of an anti‐inflammatory activity of Tregs in this phase.

TNF‐knockout mice have decreased frequency of Tregs in both thymus and spleen, compared with wild‐type, even though the underlying mechanisms remain unexplained [24]. The TNF‐TNFR2 pathway also plays anti‐inflammatory roles in pathological contexts. Indeed, the Treg‐intrinsic TNFR2 signaling provides protection against CNS autoimmunity [45, 46]. TNFR2 signaling maintains epigenetic stability of Tregs, preventing their conversion into pathogenic cells, and is required for arthritis resolution [47]. Also, in the induction phase of arthritis, T cell‐derived TNF is protective against arthritis development through the control of autoreactive T cell responses [48]. Whether Tregs were a relevant source of the “protective” TNF was not addressed in that setting.

In apparent contradiction with the evidence of TNF as a driver of Treg immune suppression, several studies have reported that, in RA patients, anti‐TNF therapy leads to the expansion and/or function improvements of Tregs [49, 50, 51, 52]. However, this effect was related to the ability of an anti‐TNF antibody (Adalimumab) to bridge monocyte‐membrane TNF and Treg‐expressed TNFR2 [11], again in favor of the TNF‐TNFR2 signal as a pro‐Treg and anti‐inflammatory axis.

Mechanistically, TNFR2 signals cooperate with other members of the TNFR superfamily in promoting NFkB activation, which drives an effector Treg program [8, 53]. In sepsis, in line with our results, TNFR2^+^ Tregs not only possess the effector immunosuppressive functions but also express cell cycle and antiapoptotic genes that support their proliferation and maintenance [38]. A metabolic remodeling may sustain the increased fitness of TNFR2^+^ Tregs. Indeed, TNFR2 costimulation induces, specifically in Tregs, an mTOR‐driven circuit of glycolysis, TCA cycle, and fatty acid synthesis [14, 15, 54]. This leads to their differentiation into tissue‐resident effector Tregs, endowed with superior migration ability [55].

Several reports point to a role for the TNFR2 axis in tumor‐associated Treg expansion and suppression [2]. In mice, exogenous TNF injection enhanced tumor growth in the lung through the expansion of pulmonary Treg via the interaction with TNFR2 [56]. Conversely, TNF blockade with a sTNFR2‐Fc improved tumor therapy in mouse models [13]. In mouse models of pancreatic cancer, TNFR2 blockade decreased tumor growth by selectively targeting effector Tregs [57]. In patients with malignant pleural effusion, a higher expression of TNFR2 on tumor‐infiltrating Tregs correlated with a worse prognosis [58]. Lactate, which is abundant in the tumor microenvironment, is imported into Tregs and promotes TNFR2 expression through histone lactylation and NFkB transcription [58].

More elusive is the source of TNF driving Treg expansion in the tumor microenvironment. TNF is considered a protumor cytokine, and TNF knockout mice generally display less aggressive tumors [2]. TNF produced by cells of bone‐marrow origin was responsible for significantly delaying mammary tumor growth in a mouse model [59]. Myeloid cells and activated effector T cells are considered relevant TNF sources. However, our results suggest that, in the tumor microenvironment, the frequency of Tregs, including TNF^+^ Tregs, increases compared with the spleen; thus, Tregs may contribute to the pool of intratumoral TNF. Also, considering that tmTNF preferentially engages TNFR2, proximity between TNFR2^+^ Tregs and cellular sources of TNF, including Tregs themselves, should favor their activation.

When TNFR2^+^ and TNFR2^−^ Tregs are cocultured, the TNFR2^+^ Tregs transfer their fluorescent dye to the TNFR2^−^ in a unidirectional fashion. Concomitantly, the TNFR2^−^ Tregs increase their Foxp3 and TNFR2 expression. We could not demonstrate that such an event is dependent on an active process of cell‐to‐cell communication and cellular material transfer, or rather due to the passive acquisition by recipient TNFR2^−^ cells of excess dye released by the TNFR2^+^ cells. However, the ability of Tregs to exchange cellular material with other cells has already been documented. Tregs are capable of performing trogocytosis of costimulatory ligands [60] and MHC II [61] from the surface of antigen‐presenting cells. The ability of Tregs to release extracellular vesicles (EVs) with an immunosuppressive cargo, and especially microRNAs, has been described in several studies [62]. Okoye et al. [63] have demonstrated that Tregs are particularly efficient in releasing EVs containing microRNAs that induce Tconvs to acquire Treg‐related genes, including Foxp3. Tregs can establish an intercellular transfer of cytoplasmic material also through the formation of gap junctions. This mechanism is involved in the transfer of cyclic AMP from Tregs to target cells [64]. Interestingly, cAMP promotes Foxp3 expression through the recruitment of the cyclic‐AMP response element binding protein on regulatory regions in the Foxp3 gene [65, 66]. Whether the ability to transfer cellular material to target cells is a peculiar feature of effector TNFR2^+^ Tregs remains an open question. The antagonistic relation between TNF and TNFR2 expression was manifested at the individual cell level, but not at the subpopulation level. Indeed, in vitro, TNFR2^+^ did not suppress the proliferation of TNF^+^ Tregs but rather increased their activation. In vivo, in the tumor microenvironment, TNFR2^+^ and TNF^+^ Tregs expanded in parallel. Taken together, our data support the hypothesis that tumors take advantage of two dependent mechanisms, namely (i) TNF production by various types of tumor‐infiltrating immune cells, favoring tumor growth per se, and (ii) the interplay between (tm)TNF and TNFR2^+^ Tregs with high suppressive activity, making the tumor microenvironment hostile against the antitumor immunity.

The TNFR2^+^ Tregs displayed a superior suppressive function in vitro. We cannot formally exclude that this may be due to a stronger suppression on a per‐cell basis. However, we could observe a higher recovery of TNFR2^+^ Tregs, which was related not to higher proliferation but rather to better survival. Gene expression analysis revealed a loss of oxidative stress signature in TNFR2^+^ Tregs, which may account for their maintenance. Indeed, TNFR2^+^ Tregs were more resistant than TNFR2^‐^ Tregs to the pretreatment with the ROS inducer menadione, which compromised survival and suppressive function. Notably, TNFR2^+^ exhibited a lower intracellular content of total ROS both in vitro and ex vivo from the tumor microenvironment.

In conclusion, our study suggests the hypothesis that the TNF‐TNFR2 signal may sustain Treg expansion and suppressive function not through a pure autocrine mechanism, where the same cell releases and senses TNF. Rather, within the Treg pool, two subpopulations exist that preferentially and stably express TNFR2 or TNF, and which cooperate in enforcing Treg suppression.

## 4 Data Limitations and Perspectives

MiR‐146a was strongly upregulated in TNFR2^+^ compared with TNFR2^−^ Tregs; however, its silencing induced a very modest rescue of TNF production in the TNFR2^+^ cells. Therefore, its actual relevance in the posttranslational regulation of TNF in these cells remains to be determined. Moreover, we did not verify whether miR‐146a‐silenced TNFR2^+^ cells lose their advantage in survival or suppressive function.

TNFR2^+^ and TNFR2^−^ Tregs have different functions, phenotypes, and gene expression profiles. Some of their features display a certain degree of stability ex vivo. However, we did not determine whether they exist along a differentiation continuum or descend from distinct precursors. We observed that TNFR2^−^ Tregs can differentiate into TNFR2^+^ Tregs upon activation, and that TNFR2^+^ Tregs display signatures of stability and strong suppressive function. Hence, we speculate that the TNFR2^−^ and TNFR2^+^ represent sequential activation states; still, the TNFR2^+^ Tregs stabilize their program. However, this hypothesis needs to be confirmed by lineage‐tracing experiments.

In the coculture of TNFR2^+^ and TNFR2^−^ Tregs, the TNFR2^−^ increase their expression of Foxp3 and TNFR2 and gain some fluorescent dye from the TNFR2^+^ Tregs. However, our data do not demonstrate whether such dye transfer is due to an active and specific process of intercellular material transfer, or to dye leakage from apoptotic cells. Moreover, we could not prove a causal link between intercellular transfer and TNFR2^−^ Treg activation, which may be simply due to a phenotypic conversion when exposed to TNFR2^+^ Treg‐derived, either soluble or membrane‐bound, factors.

Our data underscore that, when TNFR2^+^ and TNFR2^−^ Tregs are pretreated with menadione, both survival and suppressive function are reduced, especially in TNFR2^−^ Tregs. This experiment demonstrates a causal link between ROS induction and Treg survival and function. However, the higher survival of TNFR2^+^ Tregs may not fully explain their advantage in suppressive function. Indeed, TNFR2^+^ and TNFR2^−^ Tregs also display qualitative differences, as highlighted by the expression of markers that are strongly related to their stability and function.

## 5 Materials and Methods

### 5.1 Mice

C57BL/6J mice were purchased from Charles River. *Foxp3* ^CreYFP^ (stock 028363) and *Foxp3* ^eGFP‐Cre‐ERT2^ (stock 016961) mice were purchased from the Jackson Laboratory. Both male and female mice were used at 8–12 weeks of age. All mice were bred and maintained under conventional conditions at the animal facility of the Dipartimento di Scienze Anatomiche, Istologiche, Medico legali e dell'Apparato locomotore (SAIMLAL), in Sapienza Università di Roma, under protocols approved by the Italian Ministry of Health (authorization no. 1127/2020‐PR and 567/2023‐PR).

### 5.2 Collagen‐Induced Arthritis

CIA was induced in 8‐ to 12‐week‐old female C57BL/6J mice with 100 µg of denatured type II chicken collagen (Sigma Aldrich) emulsified in complete Freund's adjuvant [67]. Mice were sacrificed after 2 (acute CIA) or 10 weeks (chronic CIA), and draining lymph nodes were collected. Lymph nodes were mechanically dissociated, and single‐cell suspensions were freshly analyzed by flow cytometry.

### 5.3 Tumor Models

The 18.5 c‐myc H‐Ras V12 p53‐/‐ (hereafter “18.5”) cell line of immortal transformed hepatoblasts, inducing tumors with histological subtypes and markers characteristic of human hepatocellular carcinoma [68], was kindly provided by Micol Ravà (European Institute of Oncology, Milan, Italy). The MC38 colon adenocarcinoma cell line was kindly provided by Mario P. Colombo (Fondazione IRCCS Istituto Nazionale Tumori, Milan, Italy). Tumor cell lines were cultured in vitro for 3 to 4 passages in complete Dulbecco's modified Eagle medium (DMEM) with high glucose (Gibco) supplemented with 10% fetal bovine serum (FBS, Gibco), 2 mmol/L l‐glutamine (Sigma‐Aldrich), penicillin/streptomycin, nonessential amino acids, sodium pyruvate (Euroclone), 50 µmol/L β‐mercaptoethanol (Sigma‐Aldrich), and 10 mmol/L Hepes (Aurogene) at 37°C in a humidified 5% CO_2_ atmosphere. To obtain transplanted tumors, 10^6^ cells of the 18.5 cell line, or 5 × 10^5^ of MC38 cells, were s.c. injected into the middle flank, and mice were sacrificed after 2–3 weeks. All the in vivo experiments were authorized by the Italian Ministry of Health and performed in accordance with the institutional animal care and use committee (Organismo preposto al Benessere Animale, SAIMLAL Department, Sapienza University of Rome) and the national law (Dlgs 26/2014).

### 5.4 Lymphocyte Extraction

Splenocytes were obtained by mechanical dissociation, followed by incubation with ACK lysis solution (Gibco) for 4 min at 4°C for erythrocyte lysis. Inguinal and mesenteric lymph nodes were processed by mechanical dissociation.

To extract mononuclear cells, murine livers, lungs, and tumors were mechanically dissociated in PBS 2% FBS onto a 70‐µm cell strainer (Falcon), and a single‐cell suspension was obtained. Then, leukocytes were enriched through a 40/80 Percoll (GE Healthcare) density gradient, collecting cells at the interface between 40% and 80% Percoll solutions.

Bone marrow cells were collected from naïve mice by flushing the tibias and femurs with PBS after removing the muscles and cutting off each epiphysis. The obtained cell suspension was filtered through a 70‐µm cell strainer, and erythrocyte lysis was performed with ACK lysis solution (Gibco) for 4 min at 4°C.

### 5.5 Treg and Tconv Isolation and Culture

CD4^+^CD25^+^ (Treg) and CD4^+^CD25^−^ (Tconv) were immunomagnetically isolated from splenocytes using the CD4^+^CD25^+^ Regulatory T Cell Isolation Kit, mouse (Miltenyi Biotec).

For YFP^+^ and YFP^‐^ cell sorting, CD4^+^ T cells were first enriched from the spleens of *Foxp3* ^CreYFP^ mice using the CD4^+^ T Cell Isolation Kit, mouse (Miltenyi Biotec), stained with Fixable viability dye eFluor780 (Thermofisher Scientific), and then surface‐stained with combinations of the following Abs: CD4 BB700 or PerCP‐Cy5.5 (BD Biosciences), and TNFR2 Bv421 or PE (clone TR75‐89, BD Biosciences).

YFP^+^ cells were sorted based on TNFR2 expression as double positive (YFP^+^/TNFR2^+^) or negative cells (YFP^+^/TNFR2^−^) using a FACSAriaIII (BD Biosciences) equipped with 488, 561, Near UV375, and 633 nm lasers and FACSDiva software (BD Biosciences version 6.1.3), and retained on ice. To reduce stress, cells were isolated in gentle FACS‐sorting conditions using a ceramic nozzle of size 100 µm, a low sheath pressure of 19.84 pound‐force per square inch (psi) that maintains the sample pressure at 18.96 psi, and an acquisition rate of a maximum of 1500 events/s. Prior to culturing, an aliquot of each FACS‐sorted sample was acquired at the instruments to assess purity that was close to/higher than 95%.

In some experiments, sorting was performed on the MoFlo Astrios‐EQ flow cytometer (Beckman Coulter), equipped with four laser lines (405, 488, 561, and 642 nm) and 15 fluorescence parameters. Sorting was performed using a 100 µm nozzle tip, the sheath pressure was maintained at approximately 30 psi, and the acquisition speed was set at around 5000 events per second, to reduce the abort rate and increase sorting efficiency. The instrument acquisition software was Summit v6.3.1.

To obtain GFP^+^ TNFR2^+^ and TNFR2^−^ Tregs, CD4^+^ T cells were first immunomagnetically enriched, as detailed above, from the spleens of *Foxp3* ^eGFP‐Cre‐ERT2^ mice, then stained with Fixable viability dye eFluor780, CD4 BB700, and TNFR2 PE, and sorted on Aurora CS equipped with 405, 488, and 633 nm lasers, using an 85 µm nozzle (Cytek Biosciences).

### 5.6 In Vitro Functional Assays

For the TNF neutralization assay, spleens and tumors from C57BL/6J mice were processed as described above. CD4^+^CD25^+^ (Treg) and CD4^+^CD25^−^ (Tconv) were immunomagnetically isolated from splenocytes using the CD4+CD25+ Regulatory T Cell Isolation Kit, mouse (Miltenyi Biotec), and then labeled with the proliferation tracer CFSE (Thermofisher Scientific). Cells were cultured in vitro for 3 days with aCD3 (1 µg/mL, BD Biosciences) and irradiated splenocytes at 1:1 ratio, in the presence of anti‐TNF neutralizing Ab (BioLegend) or isotype control IgG (BioLegend), and then analyzed by flow cytometry, after 4 h restimulation with Cell Stimulation Cocktail plus cytokine transport inhibitors (Thermofisher Scientific) for intracellular TNF detection and for surface TNFR2 detection.

For Treg and Tconv in vitro cocultures, stained and sorted TNFR2^+^ Tregs, TNFR2^−^ Tregs, and Tconvs were obtained as detailed above. TNFR2^+^ Tregs were labeled with eF670 proliferation dye (eBioscience), while TNFR2^−^ Tregs and Tconvs with CellTrace Violet (CTV) (Thermofisher Scientific), and then cultured for 4 days with coated aCD3/aCD28 (1 µg/mL, BD Biosciences) and IL2 (10^3^ U/mL, Novartis).

To perform the in vitro suppression assay, sorted CD4^+^YFP^+^ TNFR2^+^ and TNFR2^−^ Treg cells were labeled with the proliferation dye eFluor 670 (10 µM, eBioscience) and separately cultured in vitro either alone or at a scaled ratio with CellTrace Violet (CTV)‐labeled CD4^+^YFP^−^ target cells (10 µM, Invitrogen) in the presence of irradiated splenocytes and soluble anti‐CD3 (1 µg/mL, BD Biosciences). After 4 days of culture, Treg survival was evaluated by comparing the frequency and the count of TNFR2^+^ and TNFR2^−^ Tregs in the gate of eFluor670^+^ cells in the different culture conditions. The suppressive capacity of Tregs was evaluated by the detection of the CTV dilution in terms of geometric mean fluorescence intensity (gMFI), and the percentage of proliferating cells in the gate of CTV^+^ cells. In some experiments, Treg were pretreated with 2.5–5 µM menadione at 10^6^cells/mL in complete medium for 1 h at 37°C, and then extensively washed before assay.

### 5.7 Flow Cytometry

For surface staining, cells were labeled with the Fixable viability dye eFluor780 (Thermofisher Scientific), and then freshly surface‐stained with combinations of the following Abs: CD4 Bv605, BB700, PerCP‐Cy5.5, or Bv785 (BD Biosciences), TCRβ Bv510 (BD Biosciences), CD3 BB700 (BD Biosciences), CD44 Bv510 (BD Biosciences), CD71 Bv605 (BD Biosciences), CD25 Bv510 (BioLegend), OX40 Bv421 (Biolegend), TNF PE‐Cy7 (to detect tmTNF, clone MP6‐XT22, BioLegend), TNFR1 APC (clone 55R‐286, BioLegend), and TNFR2 Bv421 or PE (clone TR75‐89, BD Biosciences).

For the analysis of intracellular cytokine content, cells were restimulated for 4 h in the presence of Cell Stimulation Cocktail plus cytokine transport inhibitors (Thermofisher Scientific), labeled with viability dye, and surface‐stained as detailed above, then fixed and permeabilized with BD Cytofix/Cytoperm fixation/permeabilization kit according to the manufacturer's instructions (BD Biosciences). Finally, cells were intracellularly stained with combinations of Abs to TNF PE‐Cy7 (to detect total TNF, clone MP6‐XT22, BioLegend), IFNγ Bv421 (BD Biosciences), IFNγ Bv711, IL‐17 Bv510, and IL‐10 APC (all BioLegend).

For combined cytokine and Foxp3 staining, cells were restimulated and surface‐stained as above, then fixed and permeabilized with Foxp3 transcription factor buffer set (Thermofisher Scientific), and intracellularly stained with Abs to Foxp3 PE‐eF610 (clone FJK‐16s, Thermofisher Scientific), TNF PE‐Cy7 (BioLegend), and IFNγ Bv421 (BD Biosciences).

For phenotypical analysis, sorted GFP^+^ TNFR2^+^ and TNFR2^−^ Tregs were surface‐stained with CD25 APC‐Fire810 (BioLegend), CD39 Bv605 (BD Biosciences), and CD304 Bv480 (BD Biosciences). After fixation/permeabilization, cells were intracellularly stained with Foxp3 eF450 (Thermofisher Scientific), APC‐R700 (BD Biosciences), and Helios APC (clone 22F6, Thermofisher Scientific).

For human Treg analysis, peripheral blood mononuclear cells (PBMCs) were isolated from the peripheral blood of healthy human donors using density gradient centrifugation through Lympholyte (Cedarlane) and restimulated for 4 h in the presence of Cell Stimulation Cocktail plus cytokine transport inhibitors (Thermofisher Scientific). After labeling with viability dye, cells were stained with Abs to CD14 APC‐eF780 (dump channel, Thermofisher Scientific), CD4 Bv785 (BioLegend), CD45RA Bv605 (BioLegend), CD127 PE‐Cy7, then fixed and permeabilized with Foxp3 transcription factor buffer set (Thermofisher Scientific), and intracellularly stained with Abs to FOXP3 PerCP‐Cy5.5 (clone PCH101, Thermofisher Scientific) and TNF Bv421 (clone Mab11, BioLegend).

For Primeflow analysis, CD4 cells were isolated from the spleen of a *Foxp3* ^CreYFP^ mice, stained, and then sorted as TNFR2^+^, TNFR2^−^ Tregs, and Tconvs. After 4 h restimulation with Cell Stimulation Cocktail plus cytokine transport inhibitors (Thermofisher Scientific) for intracellular TNF detection, we performed PrimeFlow using PrimeFlow RNA assay kit (eBioscience, cat# 19363) to detect TNF mRNA (QuantiGene Probe TNF‐α mRNA—AF647) together with TNF protein (TNF PE‐Cy7, BioLegend). For the Primeflow analysis of miR‐146a, the QuantiGene Probe miR‐146a‐APC was used, with β‐actin as housekeeping positive control and miR‐146a fmo (fluorescence minus one) as negative control.

ROS production was measured by flow cytometry using the CellROX Deep Red Flow Cytometry Assay Kit, according to the manufacturer's instructions (Molecular Probes, Thermofisher Scientific). Briefly, lymphocytes extracted from spleens or tumors were incubated for 45 min at 37°C with 1 µM of CellROX in complete RPMI medium, then washed and stained with Fixable Viability Dye eFluor780 and afterwards with antibodies to CD4 Bv785 and TNFR2 PE. In some experiments, cells were previously cultured in complete RPMI medium in the presence or absence of soluble CD3 (1 µg/mL, BD Biosciences) for 18 h.

Cells were acquired with an LSR Fortessa cytometer (BD Biosciences) or a Northern Lights spectral cytometer (Cytek Biosciences) and analyzed with FlowJo software version 10.8.0 (BD Biosciences).

### 5.8 ELISA

TNF concentration was measured with BD OptEIA Mouse TNF ELISA Kit (BD Biosciences, cat#560478) on supernatants of cultured TNFR2^+^ Tregs, TNFR2^−^ Tregs, and Tconvs from *Foxp3* ^CreYFP^ mice, after 4 h restimulation with Cell Stimulation Cocktail, without any cytokine transport inhibitor (Thermofisher Scientific).

### 5.9 RNA extraction, qRT‐PCR, and RNASeq

The cell pellets were frozen at −80°C in a 96‐well plate. RNA extraction was performed on the thawed pellet using the Single Cell RNA Purification Kit (Norgen Biotek). SuperScript VILO (Thermofisher Scientific) was used for cDNA synthesis. Real‐time PCR was performed using Taqman Fast Advanced Master Mix with Taqman RNA Assays (RT Primer 20X) (Thermofisher Scientific). For TNF detection, the Mm00443258_m1 Assay ID was used (Thermofisher Scientific). The relative TNF expression level was normalized to the expression of the housekeeping RNA 18S (Assay ID: Mm03928990_g1).

MicroRNAs were extracted from cells using the MicroRNA Purification Kit (Norgen Biotek). The sample was reverse transcribed using the TaqMan MicroRNA Reverse Transcription Kit and the Taqman MicroRNA Assays (RT Primer 5X) (Thermofisher Scientific) as primers. The RT‐PCR was performed using Taqman Fast Advanced Master Mix with Taqman MicroRNA Assays (TM Primer 20X) (Thermofisher Scientific). For miR‐146 detection, assay IDs 000466 and 001097 were, respectively, used for miR‐146a and miR‐146b analysis. The relative miR‐146 expression level was normalized to the expression of the housekeeping snoRNA202 using Taqman MicroRNA Assays (Assay ID: 001232). The qRT‐PCR was performed for 40 cycles of amplification with the StepOne Real Time PCR System (Thermofisher Scientific).

For RNASeq, RNA was extracted from 100,000 cells per sample with the Single Cell RNA Purification Kit (Norgen Biotek). RNA‐Seq library for mRNA sequencing was prepared with SMARTer Stranded Total RNASeq Kit—Pico Input Mammalian. All extracted RNA samples were quality‐controlled for integrity with a 2100 Bioanalyzer system (Agilent Technologies) and sequenced on a NovaSeq 6000 System (Illumina). Raw data were processed for both format conversion and de‐multiplexing by Bcl2Fastq v.2.20 of the Illumina pipeline. Read quality was evaluated using the FastQC v.0.11.8 (Babraham Institute) tool. Then adapter sequences were removed with trimgalore v.0.6.6 from FASTQ sequences using the auto detection mode and the following parameters: –clip_r1 3 –three_prime_clip_r2 3. Reads were mapped to the mouse Ensembl GRCm38 transcriptome index using salmon (v.1.3.0) [69]. Gene‐level normalization and differential gene expression analysis were performed with the Bioconductor [70] package DESeq2 v.1.28 [71] using the R environment v.4.0. To check for outliers, a regularized log transformation was applied to the count data, and a principal component analysis was generated based on genes showing the highest variance across all samples. The Wald test was used for significance testing, and the resulting FDR *p*‐values were adjusted for multiple comparisons using the Benjamini and Hochberg method [72]. Genes were considered differentially expressed genes (DEGs) only at FDR < 0.05. Gene enrichment analysis was performed by Gene Set Enrichment Analysis (GSEA) software, comparing data with previously published gene sets.

### 5.10 MiR146a Knockdown with Antagomir

Antagomirs with a cholesterol modification that allows entering cells without electroporation were used [73]. We designed antagomir to miR146a‐5p or miR146a‐3p, based on the miR‐146a sequence available on the miRBase platform (accession number: MI0000170). The antagomir sequences were the following:

miR‐146a‐5p:

[mA]*[mA]*[mC][mC][mC][mA][mU][mG][mG][mA][mA][mU][mU][mC][mA][mG][mU][mU]*[mC]*[mU]*[mC]*[mA][CHOL TEG]

miR‐146a‐3p:

[mC]*[mU]*[mG][mA][mA][mG][mA][mA][mC][mU][mG][mA][mA][mU][mU][mU][mC][mA]*[mG]*[mA]*[mG]*[mG][CHOL TEG]

The control antagomirs were the following:

Negative control 1:

[mG]*[mG]*[mU][mU][mC][mG][mU][mA][mC][mG][mU][mA][mC][mA][mC][mU][mG]*[mU]*[mU]*[mC]*[mA][CHOL TEG]

Negative control 2:

[mG]*[mA]*[mU][mA][mU][mC][mC][mC][mG][mC][mC][mG][mC][mG][mA][mU][mC][mG][mU]*[mA]*[mC]*[mC]*[mG][CHOL TEG]

Sorted lymphocytes were washed in cold PBS and then centrifuged at 300*g* for 8 min. Cells were resuspended (up to 10^7^ cells/mL) in 0.25 volume of the final culture volume of serum‐free medium. Freeze‐dried antagomir were resuspended in RNAase‐free water according to the manufacturer's instructions, to obtain a concentration of 100 µM. Samples were gently mixed and incubated with antagomir for 2 h at 5% CO_2_, 95% humidity, and 37°C. After 4 h restimulation with Cell Stimulation Cocktail plus cytokine transport inhibitors (Thermofisher Scientific), we extracted microRNAs and performed qRT‐PCR.

### 5.11 Statistical Analysis

The analysis was performed using Prism 10 (GraphPad Software) for statistical tests and graphical presentations. The statistical methods used for each analysis are described in the figure legends. Statistical significance between two or more unpaired groups of biological replicates from in vivo experiments was calculated using the nonparametric Mann–Whitney or Kruskal–Wallis test, respectively. Statistical significance between two or more unpaired groups of experimental replicates from in vitro experiments was calculated using Student's *t*‐test, one‐way, or two‐way ANOVA. In cases of multiple comparisons, *p*‐values were corrected using the Dunn, Sidak, Holm–Sidak, or Tukey methods. Data are presented as means ± SD. *p*‐values < 0.05 were considered statistically significant.

## Author Contributions

Gloria Tucci, Ilenia Pacella, Alessandra Pinzon Grimaldos, Alessandra Rossi, Ilenia Cammarata, Marta Zagaglioni, Giuseppe Pietropaolo, Valerio Licursi, Francesca Sozio, and Annalisa Del Prete performed experiments and analyzed results. Giovanna Peruzzi, Valentina Tirelli, and Massimo Sanchez provided technical support. Vincenzo Barnaba provided intellectual and material support. Silvia Piconese designed the study and performed data analysis. Gloria Tucci and Silvia Piconese wrote the first draft of the manuscript. All authors participated in the critical discussion of the results and approved the final paper.

## Ethics Statement

All mouse experiments were authorized by the Italian Ministry of Health (n° 1127/2020‐PR and 567/2023‐PR) and were performed in accordance with the institutional animal care and use committee (Organismo preposto al Benessere Animale, SAIMLAL Department, Sapienza University of Rome) and the national law (Dlgs 26/2014). Human studies were performed in accordance with the ethical guidelines of the 1975 Declaration of Helsinki and approved by the Sapienza Università di Roma—Azienda Policlinico Umberto I Ethics Committee (Prot. no. 5089).

## Conflicts of Interest

The authors declare no conflicts of interest.

## Peer Review

The peer review history for this article is available at https://publons.com/publon/10.1002/eji.70062.

## Supporting information




**Supporting File 1**: eji70062‐sup‐0001‐SuppMat.pdf.

## Data Availability

The data that support the findings of this study are available from the corresponding author upon reasonable request. The RNA‐Seq data accompanying this study are openly available through NCBI's Gene Expression Omnibus (GEO) repository, under accession number GSE291595, located at https://www.ncbi.nlm.nih.gov/geo/query/acc.cgi?acc=GSE291595.
