# Supplementary material for: TNF Production or TNFR2 Expression Characterize Distinct States of Regulatory T Cells that Cooperate in Treg Expansion in Cancer and Chronic Inflammation
Source: Eur J Immunol. 2025 Sep 21;55(9):e70062. doi: 10.1002/eji.70062 (PMC12451257; doi:10.1002/eji.70062)
Supplement: Supplementary file 1 — Supporting File 1: eji70062‐sup‐0001‐SuppMat.pdf. [file EJI-55-e70062-s001.pdf]

Supplementary figures and legends

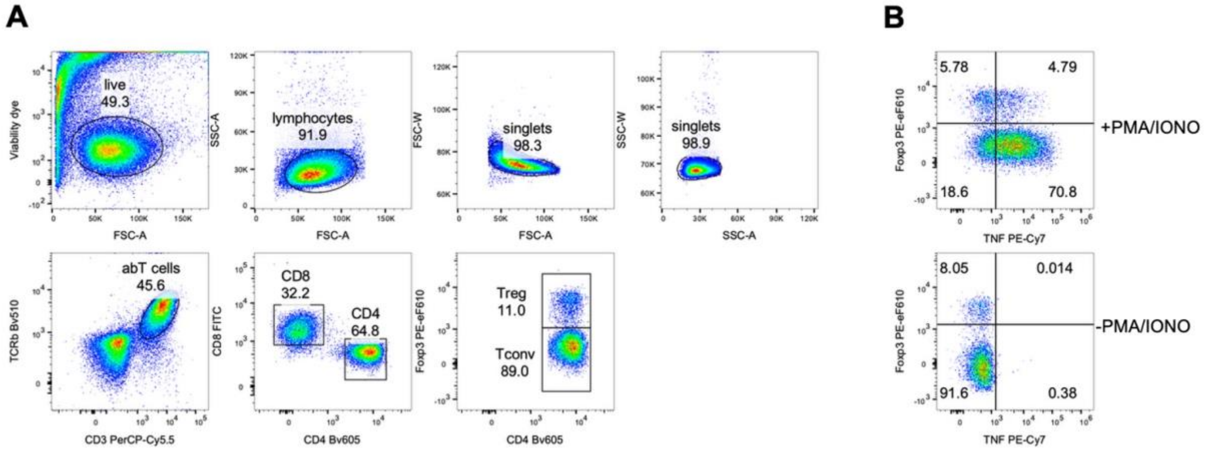

Suppl. Figure 1. Gating strategy for the identification of TNF$^+$ Tregs

A) Representative gating strategy for the identification of Tregs in a spleen sample.

B) Representative cytograms showing TNF versus Foxp3 expression in splenocytes stimulated in vitro 4 hours with or without PMA/ionomycin.

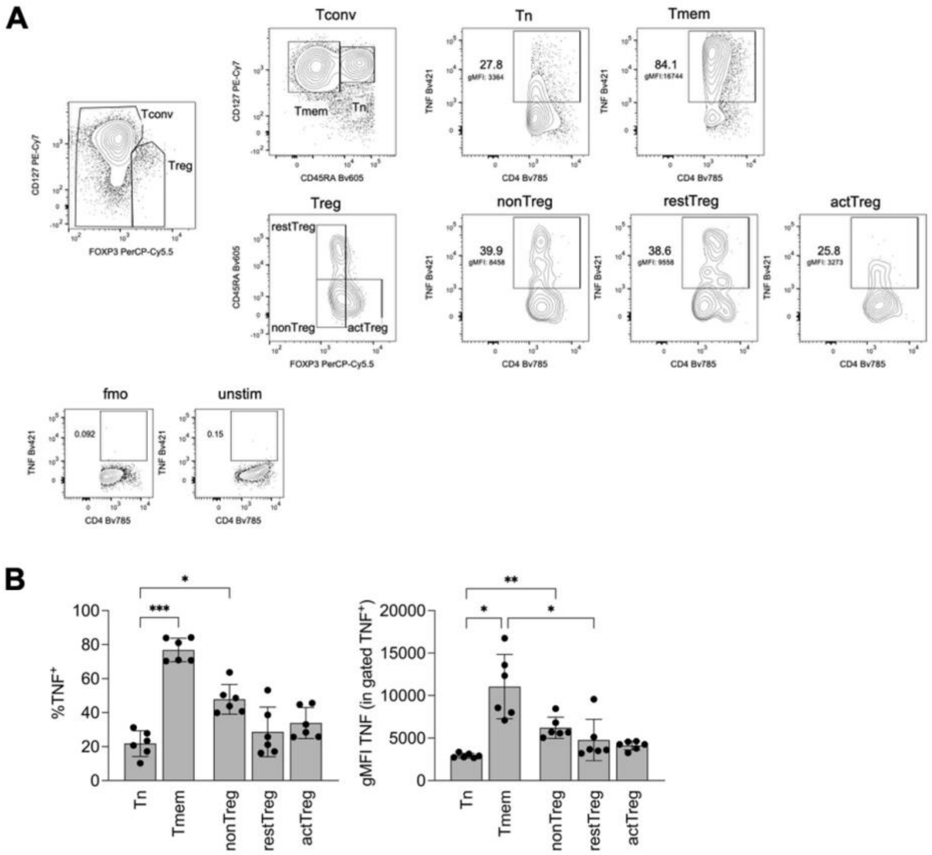

Suppl. Figure 2. Human circulating Tregs produce TNF ex vivo

A) Representative flow cytograms showing percentages of TNF^+ cells, and geometric mean fluorescence intensity (gMFI) of TNF in gated TNF^+ cells, in the indicated CD4 subsets from the peripheral blood of a human healthy donor. Fmo, fluorescence-minus-one control. Unstim, control stimulated with cytokine transport inhibitors only.

B) Cumulative analysis from samples of 6 donors, analyzed in two independent experiments. Bars represent means and SD. * *P*<0.05, ** *P*<0.01, *** *P*<0.001, by one-way ANOVA with Geisser-Greenhouse correction and Sidak multiple comparisons test.

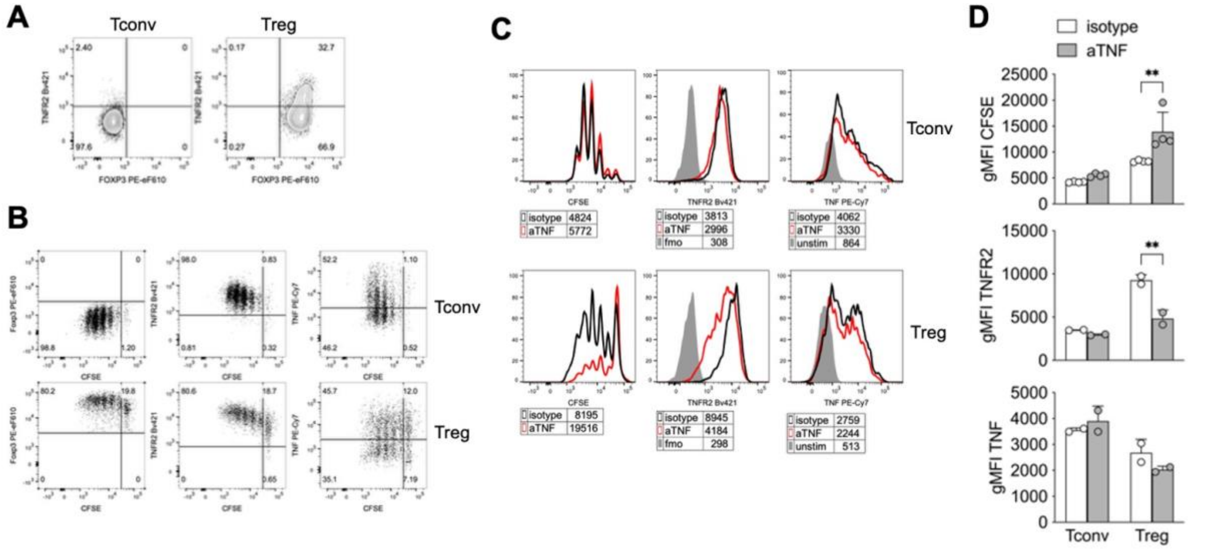

Suppl. Figure 3. TNF promotes TNFR2[+] Treg expansion in vitro

Treg and Tconv were immunomagnetically isolated from mouse spleens, labeled with CFSE, and cultured in vitro for 3 days with aCD3 and irradiated splenocytes, in the presence of anti-TNF neutralizing Ab or isotype control, and then analyzed by flow cytometry (after 4 hrs restimulation for intracellular TNF detection).

A) Representative plots showing TNFR2 expression by Tconvs and Tregs before culture.

B) Representative cytograms of Tconv and Treg cultures in control conditions.

C-D) Cytograms (B) and cumulative analysis (C) showing the gMFI of CFSE, TNFR2, and intracellular TNF, in the indicated cell types and conditions. Each condition was tested in 2-4 replicates. Bars represent means and SD. ** $P<0.01$, by two-way ANOVA with Sidak correction for multiple comparisons.

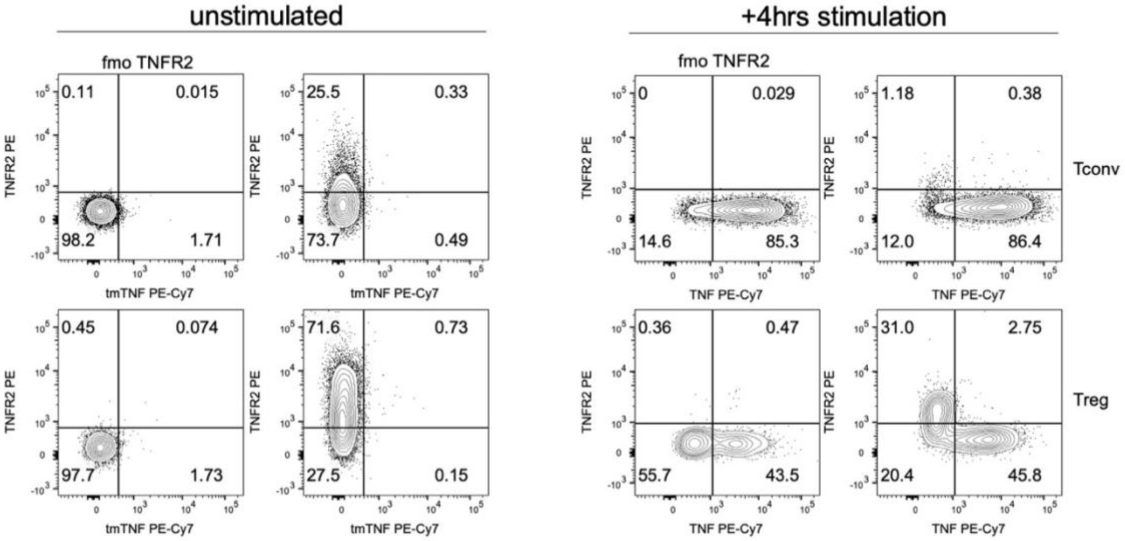

Suppl. Figure 4. In vitro restimulation affects TNFR2 detection

Representative data showing the expression of TNFR2 versus tmTNF (surface-stained) or TNF (intracellularly stained) in unstimulated or stimulated cells, respectively, in gated Tconv and Treg from the spleen of a naïve mouse (representative of 5).

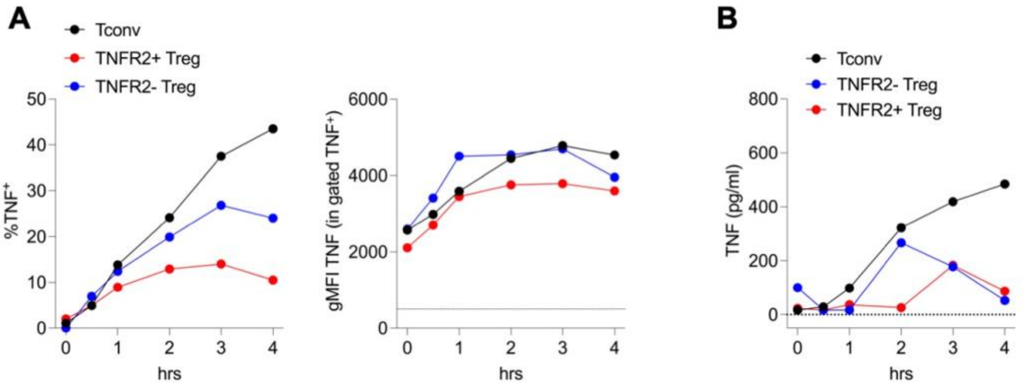

Suppl. Figure 5. Time-dependent production and release of TNF

TNFR2$^+$ and TNFR2$^-$ Treg and Tconvs were sorted as YFP$^+$ and YFP$^-$ CD4 T cells respectively, from the spleens of *Foxp3*^{CreYFP}, mice and restimulated in vitro with Cell stimulation cocktail with (A) or without (B) cytokine transport inhibitors, for 0.5, 1, 2, 3 and 4 hours.

A) Intracellular staining of TNF in the indicated subsets and conditions. Dotted line represents the fluorescence-minus-one control

B) TNF concentration in the culture supernatants as measured by ELISA. Data are from one representative of two independent experiments.

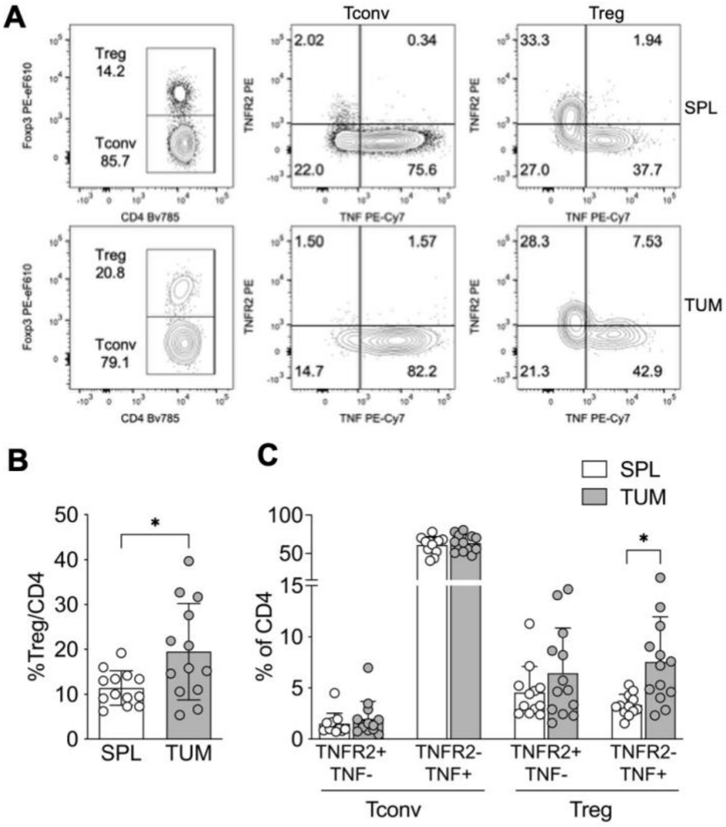

Suppl. Figure 6. TNFR2^{+}TNF^{-} and TNFR2^{-}TNF^{+} Treg can be identified also in the 18.5 tumor model

A) Representative cytograms showing Treg and Tconv frequencies and TNF/TNFR2 expression in spleen (SPL) and tumor (TUM) samples, from mice bearing 18.5 subcutaneous tumors.

B-C) Cumulative analysis showing percentages of Tregs among CD4 (B), and TNFR2^{+}TNF^{-} and TNFR2^{-}TNF^{+} Treg and Tconv among CD4 T cells (C), in SPL and TUM samples of 18.5 tumor-bearing mice (n=13). Data are pooled from two independent experiments. Bars represent means and SD. * P<0.05, by multiple Mann-Whitney test with Holm-Sidak correction for multiple comparisons.

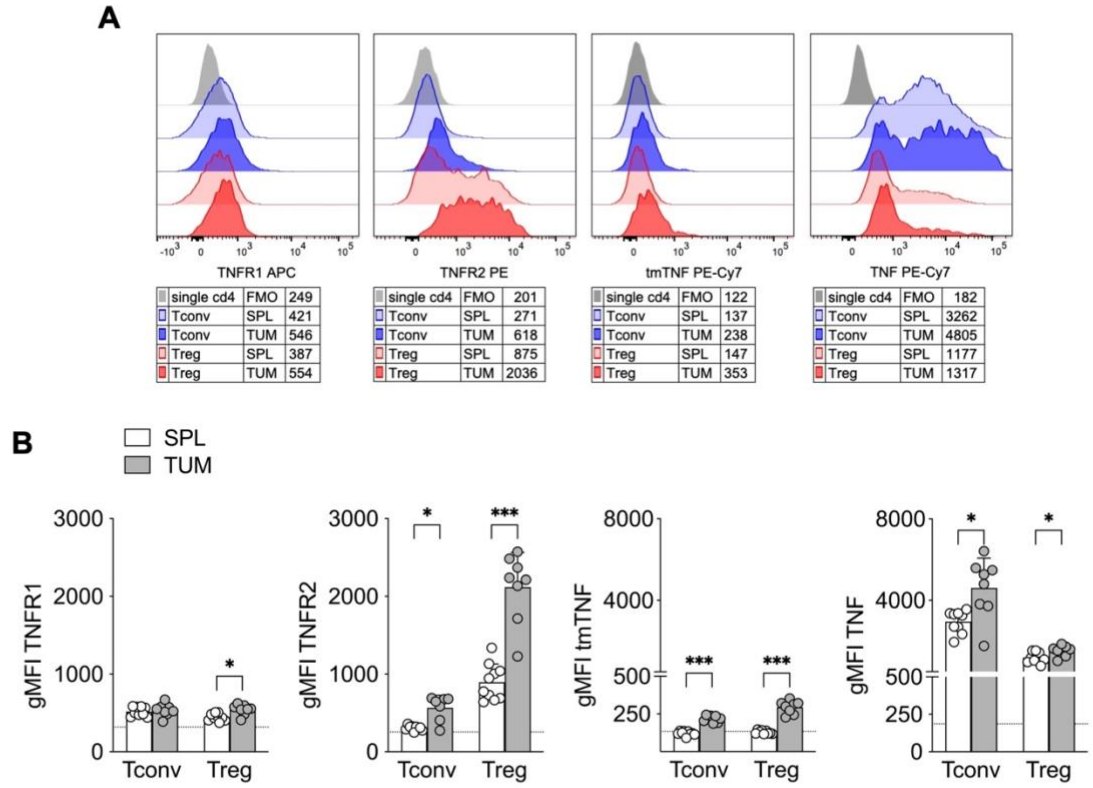

Suppl. Figure 7. TNFR1 and tmTNF are poorly expressed in Tregs and Tconvs
Analysis of the surface expression of TNFR1, TNFR2, transmembrane (tm)TNF in fresh cells, and intracellular expression of TNF in restimulated cells, in gated Treg and Tconv from SPL and TUM samples of MC38 tumor-bearing mice (n=9).

A) Representative histogram overlays. Numbers indicate the gMFI of each marker.

B) Cumulative analysis of the gMFI. The dotted lines represent the fluorescence-minus-one (FMO) control for each marker. Bars represent means and SD. * $P<0.05$, ***$P<0.001$, by multiple Mann-Whitney test with Holm-Sidak correction for multiple comparisons.

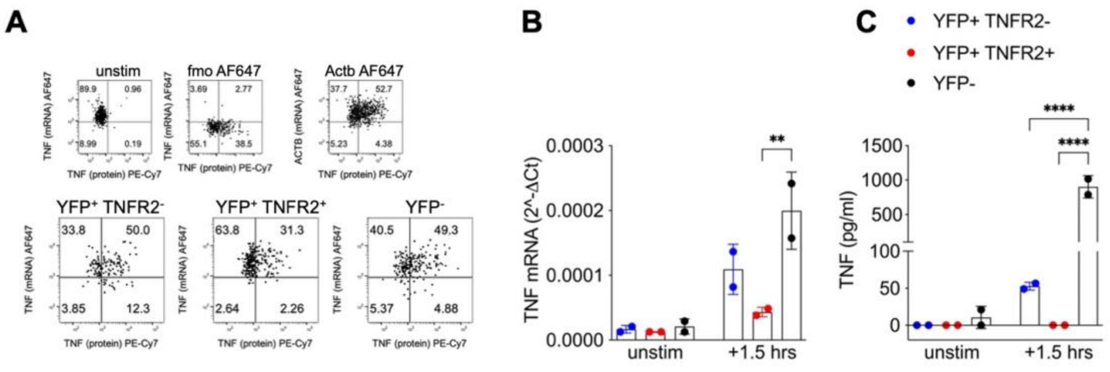

Suppl. Figure 8. TNFR2⁺ Tregs express less TNF mRNA and release less TNF protein

A) Primeflow analysis of combined TNF protein and mRNA content in the indicated cell subsets, sorted and then stimulated in vitro for 4 hours. Negative (unstimulated and fmo) and positive (Actb mRNA staining) controls for the staining are shown.

B-C) Sorted cell subsets were left unstimulated or were stimulated in vitro for 1.5 hrs with Cell stimulation cocktail without cytokine inhibitors. (D) TNF mRNA expression was analyzed by qRT-PCR and calculated as the 2^-ΔCt against the 18S gene. (E) TNF concentration was measured by ELISA in the culture supernatants. Each condition was tested in duplicates. Bars represent means and SD. ** *P*<0.01, *****P*<0.0001, by two-way ANOVA with Tukey's multiple comparison test.

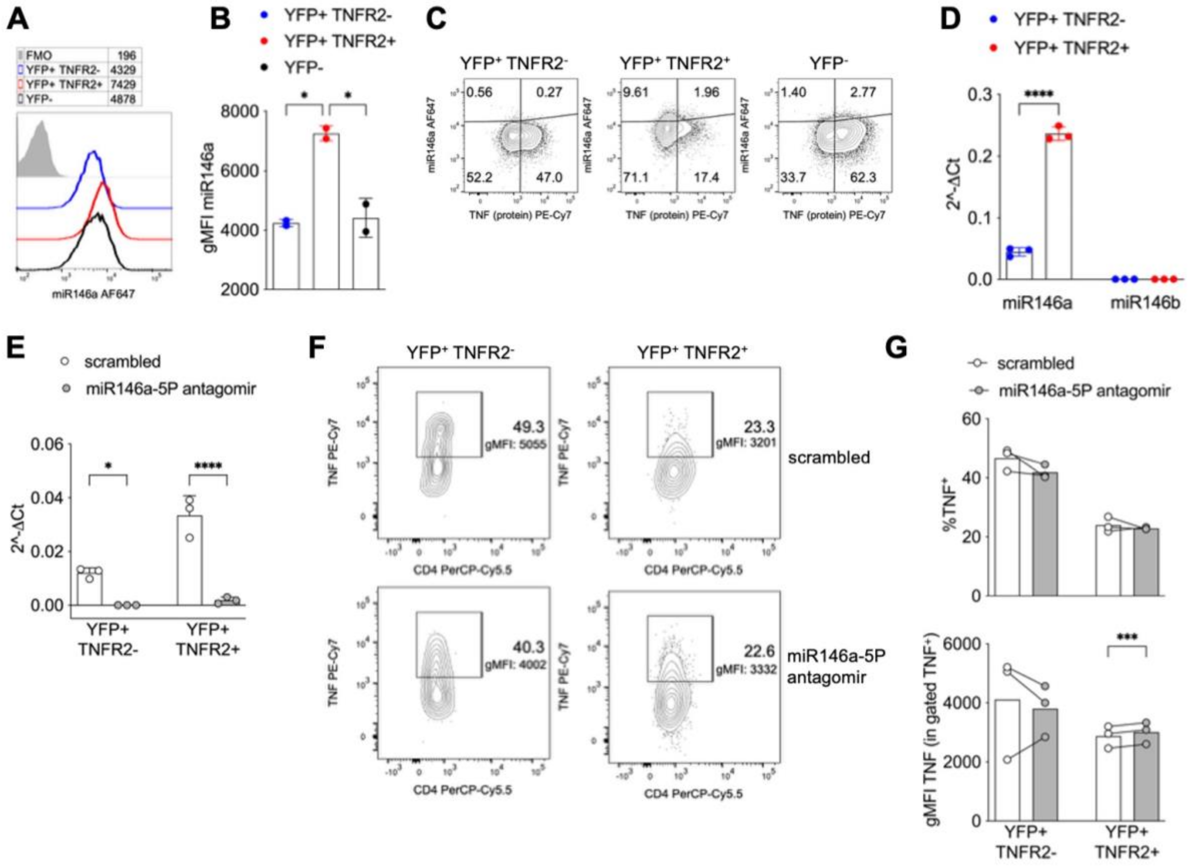

Suppl. Figure 9. MiR146a contributes to the post-transcriptional regulation of TNF in TNFR2⁺ Tregs

A-C) TNFR2$^+$ and TNFR2$^-$ Tregs and Tconvs were sorted as YFP$^+$ and YFP$^-$ CD4 T cells respectively, from the spleens of *Foxp3*^{CreYFP} mice and restimulated in vitro 4 hours before analysis of intracellular TNF and miR146a by Primeflow. Histogram overlays (A) and analysis of the gMFI (B) are shown. C) Contour plots showing TNF versus miR146a expression. Data are from one experiment representative of two. Each condition was tested in duplicates. * *P*<0.05, by one-way ANOVA with Holm-Sidak correction for multiple comparisons.

D) The expression of miR146a and miR146b was analyzed by qRT-PCR and calculated as the 2^-ΔCt against the snoRNA202 in sorted TNFR2$^+$ and TNFR2$^-$ Tregs. Data are from one representative of three independent experiments. Each condition was tested in triplicates. **** *P*<0.0001, by one-way ANOVA.

E) Sorted TNFR2$^+$ and TNFR2$^-$ Tregs were treated in vitro with miR146a-5P antagomir or scrambled control and restimulated in vitro 4 hours before analysis. Data are from one representative of three independent experiments. (E) MicroRNA were extracted and miR146a was analyzed by qRT-PCR. * *P*<0.05, **** *P*<0.0001, by two-way ANOVA with Sidak correction for multiple comparisons.

F-G) TNF production was analyzed by intracellular flow cytometry in knocked-down cells. (F) Representative flow cytograms and (G) cumulative analysis showing percentages of TNF$^+$ cells, and geometric mean fluorescence intensity (gMFI) of TNF in gated TNF$^+$ cells, in the indicated conditions, from three independent experiments. *** *P*<0.001, by two-way ANOVA with Geisser-Greenhouse correction and Sidak multiple comparisons test.

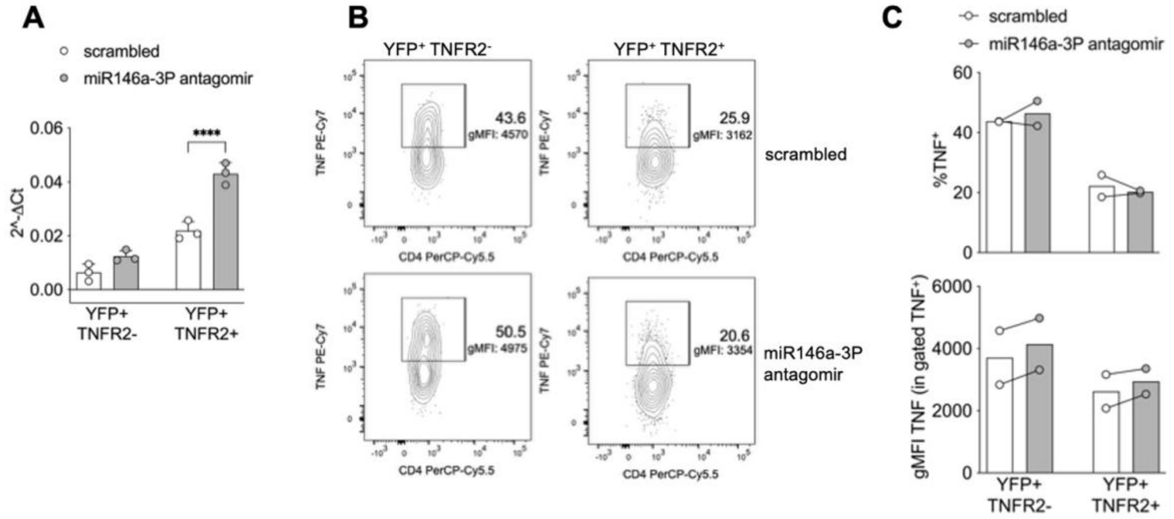

Suppl. Figure 10. miR146a-3P does not regulate expression of miR146a or TNF

Sorted TNFR2$^+$ and TNFR2$^-$ Tregs were treated in vitro with miR146a-3P antagomir or scrambled control and restimulated in vitro 4 hours before analysis. Data are from one representative of two independent experiments.

A) MicroRNA were extracted and miR146a was analyzed by qRT-PCR. **** P<0.0001, by two-way ANOVA with Sidak correction for multiple comparisons.

(B-C) TNF production was analyzed by intracellular flow cytometry. (B) Representative flow cytograms and (C) cumulative analysis showing percentages of TNF$^+$ cells, and geometric mean fluorescence intensity (gMFI) of TNF in gated TNF$^+$ cells, in the indicated conditions, from two independent experiments.

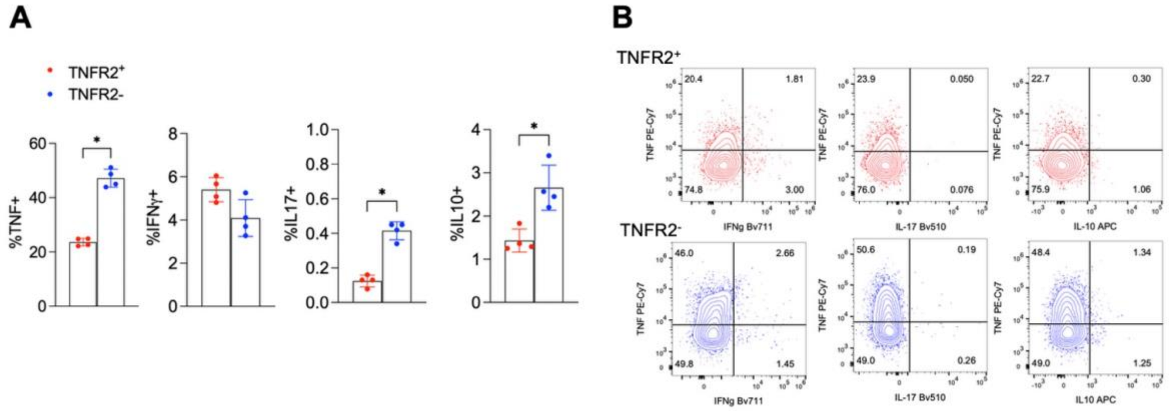

Suppl. Figure 11. TNFR2$^+$ Tregs produce less IL-17 and IL-10 than TNFR2$^-$

A-B) TNFR2$^+$ and TNFR2$^-$ Tregs were sorted from spleens of *Foxp3*^{CreGFP} mice, restimulated 4 hours with PMA/ionomycin, then intracellular staining was performed. Cumulative data (A) and representative plots of cytokine expression versus TNF (B), in gated GFP$^+$ cells, are shown. Data are from one experiment representative of two, each performed with 2-4 mice. * *P*<0.05, by Mann-Whitney test.

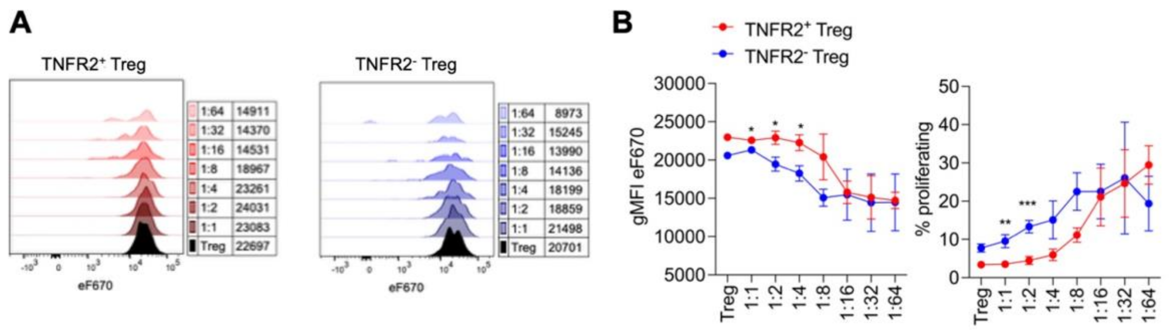

Suppl. Figure 12. TNFR2$^+$ Tregs do not proliferate more than TNFR2$^-$

Sorted TNFR2$^+$ and TNFR2$^-$ Tregs were labeled with the eFluor670 proliferation dye and cultured in vitro either alone or at scaled ratios with CTV-labelled Tconv.

A) Histogram overlays showing eF670 dilution and respective gMFI in TNFR2$^+$ (red) and TNFR2$^-$ (blue) Tregs in the indicated conditions.

B) Analysis of the gMFI of eF670 and of the percentage of proliferating cells in gated Treg in the indicated conditions.

Data are from one representative of two independent experiments. Each condition was tested in 2-6 replicates. * P<0.05, ** P<0.01, *** P<0.001, by unpaired t test with Holm-Sidak correction for multiple comparisons.

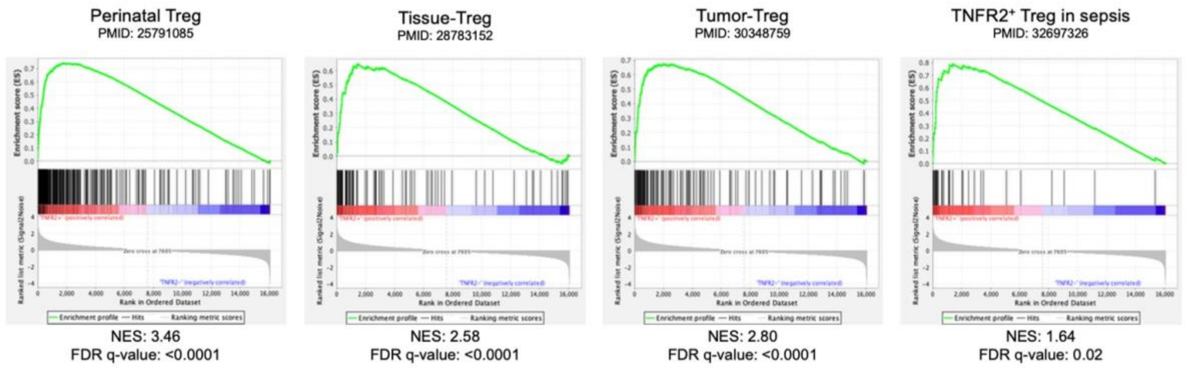

Suppl. Figure 13. TNFR2$^+$ Treg show signatures of Treg with superior suppressive function
Gene set enrichment analysis of the transcriptome of TNFR2$^+$ versus TNFR2$^-$ Treg. Gene sets were obtained from published signatures of perinatally expanded Tregs [1], tissue-Tregs [2], tumor-Tregs [3], or sepsis-associated TNFR2$^+$ Treg [4]. Normalized enrichment scores (NES) and FDR q values are shown under each plot.

1 **Yang, S., Fujikado, N., Kolodin, D., Benoist, C. and Mathis, D.,** Immune tolerance. Regulatory T cells generated early in life play a distinct role in maintaining self-tolerance. *Science* 2015. **348**: 589-594.

2 **Delacher, M., Imbusch, C. D., Weichenhan, D., Breiling, A., Hotz-Wagenblatt, A., Trager, U., Hofer, A. C., Kagebein, D., Wang, Q., Frauhammer, F., Mallm, J. P., Bauer, K., Herrmann, C., Lang, P. A., Brors, B., Plass, C. and Feuerer, M.,** Genome-wide DNA-methylation landscape defines specialization of regulatory T cells in tissues. *Nat Immunol* 2017. **18**: 1160-1172.

3 **Magnuson, A. M., Kiner, E., Ergun, A., Park, J. S., Asinovski, N., Ortiz-Lopez, A., Kilcoyne, A., Paoluzzi-Tomada, E., Weissleder, R., Mathis, D. and Benoist, C.,** Identification and validation of a tumor-infiltrating Treg transcriptional signature conserved across species and tumor types. *Proc Natl Acad Sci U S A* 2018. **115**: E10672-E10681.

4 **Gaborit, B. J., Roquilly, A., Louvet, C., Sadek, A., Tessoulin, B., Broquet, A., Jacqueline, C., Vourc'h, M., Chaumette, T., Chauveau, M., Asquier, A., Bourdiol, A., Le Mabecque, V., Davieau, M., Caillon, J., Boutoille, D., Coulpier, F., Lemoine, S., Ronin, E., Poschmann, J., Salomon, B. L. and Asehnoune, K.,** Regulatory T Cells Expressing Tumor Necrosis Factor Receptor Type 2 Play a Major Role in CD4+ T-Cell Impairment During Sepsis. *J Infect Dis* 2020. **222**: 1222-1234.
